# A Review on Damage and Rupture Modelling for Soft Tissues

**DOI:** 10.3390/bioengineering9010026

**Published:** 2022-01-10

**Authors:** Sai Naga Sri Harsha Chittajallu, Ashutosh Richhariya, Kwong Ming Tse, Viswanath Chinthapenta

**Affiliations:** 1 Micro Mmechanics Lab, Department of Mechanical and Aerospace Engineering, Indian Institute of Technology Hyderabad, Kandi 502284, India; me18resch01003@iith.ac.in; 2 Centre for Technology Innovation, L V Prasad Eye Institute, Hyderabad 500032, India; ashutosh@lvpei.org; 3 Department of Mechanical and Product Design Engineering, Swinburne University of Technology, Melbourne 3122, Australia

**Keywords:** soft tissue, damage mechanics, fracture mechanics, biomaterials, FEM

## Abstract

Computational modelling of damage and rupture of non-connective and connective soft tissues due to pathological and supra-physiological mechanisms is vital in the fundamental understanding of failures. Recent advancements in soft tissue damage models play an essential role in developing artificial tissues, medical devices/implants, and surgical intervention practices. The current article reviews the recently developed damage models and rupture models that considered the microstructure of the tissues. Earlier review works presented damage and rupture separately, wherein this work reviews both damage and rupture in soft tissues. Wherein the present article provides a detailed review of various models on the damage evolution and tear in soft tissues focusing on key conceptual ideas, advantages, limitations, and challenges. Some key challenges of damage and rupture models are outlined in the article, which helps extend the present damage and rupture models to various soft tissues.

## 1. Introduction

Soft tissue refers to non-mineralised tissue or organ in living creatures that are fibrous, which connects, support, or surrounds other structures and organs of the body [1,2,3]. Soft tissues are classified into two major groups: connective tissues and non-connective tissues. Tendons, skin, fat, ligaments fall under connective tissues, while muscles, nerves, and blood vessels fall under non-connective tissues [2,3,4]. Each tissue undergoes a specific mechanical loading according to its functionality. For example, blood vessels and arterial valves experience cyclic circumferential loading due to blood circulation; the eye tissue experiences constant tension due to intraocular pressure; the articular cartilages covering the ends of bones experience constant compression and friction.

**Figure 1:**
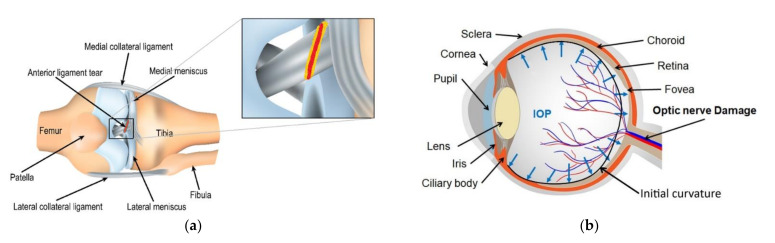
(**a**) Anterior cruciate ligament tear with the close view explaining the tear in the ligaments (**b**) Eyeball under glaucoma.

In medical sciences, the study of damage in soft tissues is essential in both physiological and pathological conditions. Damage in soft tissues is intrinsic either due to excessive external mechanical loading [5,6] or pathological condition [7,8]. A pathological condition such as glaucoma in the human eye leads to excessive accumulation of aqueous humour that causes high intraocular pressure and damages the optic nerve head, leading to vision loss (Figure 1b) [9,10]. While damage due to external mechanical loading occurs when the applied load exceeds the physiological limit of the tissue (supra-physiological load). For example, ligament tear, which athletes encounter, occurs due to excessive or repetitive tensile loading during sport (Figure 1a) [5]. During extensive physical activities, at times, ligaments in the knee undergo supra-physiological loading causing flexed knee and lateral rotation of the tibia, resulting in anterior cruciate ligament tear [5,11]. Knowledge of the damage in soft tissues would help to come up with improved clinical practices.

Understanding the biomechanics of the soft tissues is essential for modelling damage. Experimental tests provide fundamental insights into underlying deformation and damage mechanisms. Since the inception of biomechanics, tissues from animals, cadavers, and volunteer living subjects have been used extensively for experimental tests [12,13,14,15,16,17,18]. Despite providing valuable information, the experimental approach suffers several limitations. These include stringent ethical and animal certifications, limited availability of subjects and non-standard experimental procedures. In addition, the tissue responses captured in the experiments are passive, i.e., the effect of muscle cells is neglected. Moreover, the risk of tissue degradation and tissue to tissue variations are always present in the experiments [19]. Therefore, the modelling and simulation of tissue damage is a cost-effective alternative in conceptualizing the improved clinical practices, tissue engineering, and medical device development with limited experimental validation. For instance, modelling the inelastic behaviour of arteries during angioplasty would help in optimising the surgical procedure. It is possible to understand the rupture phenomena of aortic aneurysms under in-vivo conditions using rupture simulations, which would help in its diagnosis. Furthermore, rupture simulations that simulate the in-vivo loading conditions would help in designing the artificial implants.

The inelastic deformation behaviour of soft tissue depends on the stress state and extent of the damage. The stress state is determined using the material constitutive relation: it governs the displacement response of the material to the mechanical loading. While the damage is characterised using initiation and evolution laws. And the failure is captured through rupture laws. Hence, the present study discusses various soft tissue constitutive, damage, and rupture models.

Soft tissue consists of cells, elastin, collagen, and a non-mineralized ground matrix. Collagen fibres are of high stiffness compared to the rest of the constituents of the tissue, and they majorly contribute towards the overall stiffness of the tissue [20,21]. Based on their constituents and structure, soft tissues exhibit non-linear, anisotropic, hyperelastic, and viscoelastic behaviour [21]. Constitutive behaviour varies from tissue to tissue, depending on the collagen fibre distribution and orientation inside the tissue. For example, in connective tissues such as ligaments and tendons, the collagen fibre orientation is regular and unidirectional [22,23]. While in non-connective tissues, such as arterial walls, collagen fibres orientation is irregular and multi-directional [6,20,21]. In the arterial wall, collagen fibre distribution is arranged in a double-helical pattern [24]. Hence due to the tissue-specific arrangement of the collagen and other constituents, ligaments possess high tensile strength, i.e., 50–100 MPa, while the arteries possess low tensile strength, i.e., 0.3–0.8 MPa [2,23]. However, both the tissues exhibit anisotropic hyperelastic behaviour.

**Figure 2:**
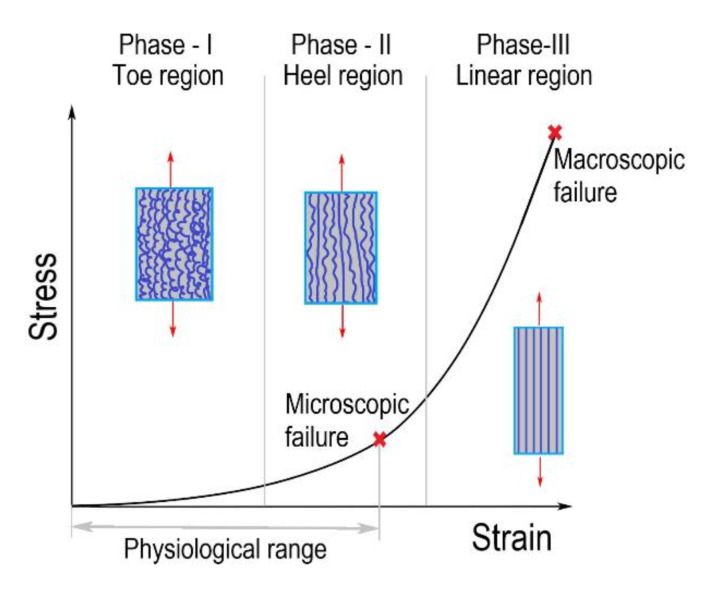
Stress-strain response of skin tissue. Adopted with permission from from ref. [2], Copyright 2001 Elsevier.

Figure 2 represents a typical stress-strain curve of skin subjected to uniaxial tension. The skin constitutes three layers: epidermis, dermis, and hypodermis [25]. Wherein the epidermis is the thin outermost cellular layer. The dermis is the middle layer with elastin and collagen fibres embedded in the extrafibrillar matrix, providing strength. The hypodermis is the innermost supportive layer that primarily consists of adipose tissue [25,26]. The skin majorly consists of collagenous fibres that account for 60 to 80% of dry weight, and the fibres are woven into a rhombic shaped pattern [2,25]. In general, most collagenous soft tissues manifest a typical J-shaped stress-strain behaviour, similar to that of the skin, as illustrated in Figure 2 [2,25].

In the current review, the mechanical behaviour of the skin is explained with a macroscopic perspective. However, the nanomechanics of collagen fibres where hydrogen and covalent bonds in the protein backbone plays a key role also contributes to the macroscopic mechanical behaviour [26,27]. The deformation behaviour of the skin can be divided into three phases:

1. The initial region of the stress-strain response, i.e., phase-I (toe region). The soft tissue’s mechanical behaviour in this region is similar to a soft isotropic rubber sheet. The collagen fibres are in a relaxed state, and they appear wavy and crimped. Therefore, very low stress is required for attaining large deformation without stretching the collagen fibres. As a result, the mechanical behaviour in phase-I is approximately linear, and the elastic modulus is low (0.1–2 MPa) [25,28].
2. In phase-II (heel region), the tissue exhibits a highly non-linear mechanical behaviour [25]. The collagen fibres get uncrimped as they elongate with the increase in the load. The elongated fibres slide into the matrix and align themselves to the direction of load, thereby increasing the load-carrying capacity.
3. In Phase-III (linear region), the tissue exhibits stiffer and linear behaviour. Most of the fibres get aligned to the loading direction; hence, no crimp pattern is observed. The aligned and straightened fibres resist the load, making the tissue stiffer and linear in mechanical behaviour [23,28]. Beyond phase III, ultimate tensile strength is reached, resulting in tissue rupture.

Damage in the soft tissues is defined as “injury or harm that reduces value or usefulness” [29], and rupture results from accumulated damage, which is catastrophic. Damage models phenomenologically capture the damage in the soft tissue due to pathological or supra-physiological conditions. On the other hand, fracture mechanics concepts alone cannot quantify the soft tissue rupture that exhibits a toughening mechanism leading to high defect tolerance [30,31]. Hence rupture in soft tissues needs a damage model along with fracture mechanics. Earlier review works [6,7,32,33] presented damage and rupture separately, wherein this paper reviews both damage and rupture in soft tissues. The earlier reported reviews are either tissue-specific or confined to phenomenological models. The current article reviews the recently developed damage models and rupture models that considered the microstructure of the tissues. In Section 2, continuum kinematics and soft tissue material constitutive model is presented that helps in reviewing the damage and rupture models in a unified manner. The damage models for soft tissues are reviewed in Section 3, which are classified into three groups, namely (1) continuum damage mechanics (CDM), (2) pseudo-elasticity, and (3) softening hyperelasticity. In Section 4, soft tissue rupture models are reviewed and classified into three groups: (1) extended finite element method (XFEM), (2) cohesive zone modelling (CZM), and (3) crack phase-field method (CPFM). A summary highlighting all the damage and rupture models and their respective challenges is discussed in Section 5, and the final concluding remarks are given in Section 6.

## 2. Kinematics and Constitutive Model

This section is the preliminary to provide a unified representation for all the damage and rupture models. The essential kinematics of the continuum mechanics needed for defining the damage, rupture and material models are presented.

**Figure 3:**
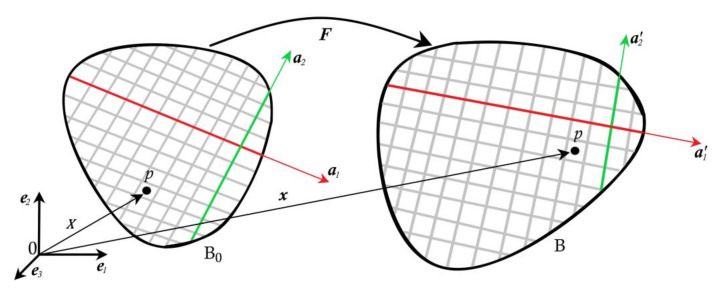
Configuration of the continuum body from reference state to the deformed state.

Figure 3 shows a body that occupies the domain $B_{0}$ in reference configuration and occupies domain B in the current configuration. Let X denote the material point in the reference configuration, and x denote the same material point in the current configuration. Deformation map $F=\frac{\partial x}{\partial X}$ maps reference configuration to final configuration and Jacobian $J=\det\left(F\right)>0$. Right Cauchy–Green tensor is $C=F^{T}F$. Modified right Cauchy–Green tensor $\bar{C}=J^{-\frac{2}{3}}C$ is used for pure distortion. $a_{1}$ and $a_{2}$ be the orientation of two fibre families in the reference configuration. $a_{1}^{\prime }$ and $a_{2}^{\prime }$ be their corresponding orientations in the current configuration. They are related as $a_{1}^{\prime }=Fa_{1}$ and $a_{2}^{\prime }=Fa_{2}$.

Invariants associated with Cauchy–Green deformation tensor are

(1) $$I_{1}=tr\left(C\right),\;I_{2}=tr\left(cof\left(C\right)\right)\;\mathrm{and}\;I_{3}=det\left(C\right)$$

Invariants related to fibres are given as

(2) $$I_{4}=C:\left(a_{1}⊗a_{1}\right)=a_{1}^{\prime }.a_{1}^{\prime },\;I_{6}=C:\left(a_{2}⊗a_{2}\right)=a_{2}^{\prime }.a_{2}^{\prime }$$

The majority of damage and rupture models reviewed have used the HGO (Holzapfel Gasser and Ogden) model for defining the material constitutive response [34]. Hence, the HGO model is briefly discussed here. In the HGO model, the isotropic and anisotropic parts of the strain energy function is decomposed as:

(3) $$\Psi =\Psi_{vol}+\Psi_{dev}+\Psi_{ani}$$

where

(4) $$\Psi_{vol}=\frac{1}{2}K{\left(J-1\right)}^{2}$$

(5) $$\Psi_{dev}=\frac{\mu }{2}\left({\bar{I}}_{1}-3\right)$$

(6) $$\Psi_{ani}=\underset{i=4,6}{\sum }\frac{k_{1}}{2k_{2}}\left[\exp\left(k_{2}\langle {\bar{E}}_{i}^{\;}\rangle {}^{2}\right)-1\right]$$

where the first two components are dilatational $\left(\Psi_{vol}\right)$ and deviatoric $\left(\Psi_{dev}\right)$ parts of the isotropic response and last component correspond contribution of the two families of fibres $\left(\Psi_{ani}\right)$. The invariants are defined for the modified right Cauchy–Green tensor. K is the bulk modulus, μ is the shear modulus, $k_{1}$ and $k_{2}$ are the parameters that define the contribution of fibres. The Macaulay bracket is defined as ($\langle ■\rangle =\left(\left|■\right|+■\right)\frac{1}{2}$) that assumes the fibres can only support tension. ${\bar{E}}_{i}^{\;}$ is a strain-like quantity that represents the deformation of the family of fibres $a_{l}\;\left(l=1,\;2\right)$ and it is defined as

(7) $${\bar{E}}_{i}^{\;}\overset{\mathrm{def}}{=}\kappa \left(\;{\bar{I}}_{1}-3\right)+\left(1-3\kappa \right)\left(\;{\bar{I}}_{i}-1\right),\;i=4,\;6$$

This model assumes that the collagen fibres orientation and distribution for the respective family of fibres, which are described along a preferred mean direction. Wherein, κ is the radial dispersion parameter that defines the radial symmetry dispersion of the fibre orientation. κ is defined based on the fibre orientation density function ρ and the fibre orientation, Θ, as

(8) $$\kappa =\frac{1}{4}\int_{0}^{\pi }\rho \left(\Theta \right)\sin^{3}\Theta d\Theta$$

The radial dispersion parameter $\;\kappa \in \left[0,\frac{1}{3}\right]$, where κ=0 describes perfectly aligned fibres without dispersion and $\kappa =\frac{1}{3}$ describes random distribution where the material behaves as isotropic. The strain, as with quantity ${\bar{E}}_{i}^{\;}$, becomes

(9) $${\bar{E}}_{i}^{\;}=\left(\;{\bar{I}}_{i}-1\right)\;\mathrm{for}\;\kappa =0$$

(10) $${\bar{E}}_{i}^{\;}=\frac{\left(\;{\bar{I}}_{1}-1\right)}{3}\;\mathrm{for}\;\kappa =\frac{1}{3}$$

Further, the anisotropic strain energy function (6) for each family of fibres can be additively decomposed as $\Psi_{ani}=\Psi_{f1}+\Psi_{f2}$ and for aligned fibres the strain energy is given as

(11) $$\Psi_{af1}=\frac{k_{1}}{2k_{2}}\left[\exp k_{2}{\left({\bar{I}}_{4}-1\right)}^{2}-1\right]$$

and

(12) $$\Psi_{af2}=\frac{k_{1}}{2k_{2}}\left[\exp k_{2}{\left({\bar{I}}_{6}-1\right)}^{2}-1\right]$$

Similarly, for distributed fibres, the strain energy density ($\Psi_{ani}=\Psi_{df1}+\Psi_{df2}$) is given as

(13) $$\Psi_{df1}=\frac{k_{1}}{2k_{2}}\left[\exp k_{2}\langle \kappa \left(\;{\bar{I}}_{1}-3\right)+\left(1-3\kappa \right){\left(\;{\bar{I}}_{4}-1\right)\rangle }^{2}-1\right]$$

and

(14) $$\Psi_{df2}=\frac{k_{1}}{2k_{2}}\left[\exp k_{2}\langle \kappa \left(\;{\bar{I}}_{1}-3\right)+\left(1-3\kappa \right){\left(\;{\bar{I}}_{6}-1\right)\rangle }^{2}-1\right]$$

## 3. Damage Models

**Figure 4:**
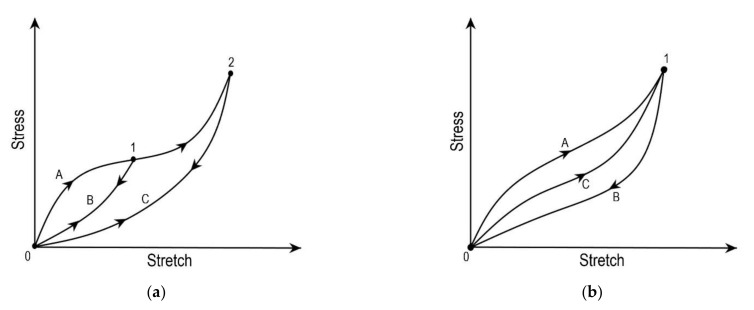
Stress vs. stretch plots describing (**a**) Idealized response of the Mullins effect where path A–B first cycle and second cycle path A–C affected by softening, (**b**) Stress softening in hysteresis, A-B first cycle and C–B second cycle affected by softening.

Damage in the soft tissues occurs when the applied load goes beyond the physiological range, as shown in Figure 2. The physiological limit of the soft tissue lies in phase-II [28]; when the applied load exceeds this limit, the damage at the microscopic level initiates, resulting in microscopic failure. Damage in a material is a progressive physical phenomenon that leads to failure, as shown in Figure 2. Experimental studies have reported four phenomena associated with the damage in soft tissues: (a) Mullins effect, (b) hysteresis, (c) permanent set, and (d) rupture [6,35]. Under the cyclic loading of soft tissue, the stress required after the first cycle for the same stretch reduces for the consecutive cycles (Figure 4a). This stress softening phenomenon in materials under cyclic loading is known as the Mullins effect [36]. The maximum load governs the Mullins effect during the loading cycles. In the literature, the Mullins effect is extensively studied by several authors on arteries [37,38], vaginal tissue [39], etc. The continuous softening of the material subjected to cyclic loading under constant load is known as hysteresis (Figure 4b). The softening phenomenon continues until a saturation point is reached [6,35,40,41,42]. Hysteresis is used to precondition the soft tissues at lower loads to overcome the shape effects in experimental tests [43,44]. Hysteresis studies are often reported for arteries by Pena et al. [39] and Balzani et al. [37,42]. The inelastic behaviour that occurs due to accumulated strain in the soft tissue due to load is known as the permanent set [35]. This phenomenon is also studied extensively for soft tissues as with arteries [45,46] and bioprosthetic heart valves [47]. Lastly, the accumulated microscopic damage in the tissue leads to a macroscopic failure known as rupture. The tissue rupture may arise due to the failure of the matrix or rupture in fibres. In the literature, rupture studies are reported in arteries [48,49], corneas [50,51], skin [52], ligaments [53], etc.

This section may be divided by subheadings. It should provide a concise and precise description of the experimental results, their interpretation, and the experimental conclusions that can be drawn.

The aforementioned phenomena require a damage model along with a constitutive response to capture the deformation behaviour of the tissue. In general, the damage models for soft tissues available in the literature can be broadly classified into three categories: (a) models based on continuum damage mechanics (CDM), (b) theory of pseudo-elasticity, and (c) the softening hyperelasticity approach [6]. The CDM gives a continuum level description for damage phenomena, i.e., it provides damage initiation, propagation, and microscopic failure [54,55]. It is based on an irreversible thermodynamics process where the Clausius–Duhem inequality is used for defining the internal state variables and internal dissipation. In CDM, the damage is modelled using the state variables that defines the onset of the damage and govern the degraded material response. For soft tissues, CDM has been used to analyse the Mullins effect, permanent set, and tissue rupture [37,56,57]. In CDM, the damage initiation is characterised based on the concept of equivalent strain for undamaged material introduced by Simo and Ju [54]. Later, Miehe used the same concept to define discontinuous damage characterised by the maximum strain in the loading path and continuous damage characterised by strain-rate-dependent [7]. Comparatively, CDM requires a higher number of material parameters to define the damage.

Phenomenologically, pseudo-elasticity models the damage in the soft tissues using two elastic material models, one during loading and another while unloading. In this approach, the stress-strain behaviour in cyclic loading is defined using fewer material parameters than CDM. The pseudo-elasticity approach has been used to model arterial walls [58]; Mullins effect in rubber [59,60]; Mullins effect and permanent set in brain tissue and arteries [17,38,61]. The softening hyperelasticity technique was introduced as a substitute to CDM and pseudo-elasticity by Volokh [62], wherein the constitutive response is incorporated with strain softening using the material constants named as energy limiters. In this approach, the internal variables to quantify damage, initiation condition, and the evolution equation for damage are not required. Therefore, it is more straightforward than CDM and pseudo-elasticity. This approach has been adopted by Li and Lou [63] in modelling human and animal skin. The present section systematically reviews the modelling techniques such as CDM, pseudo-elasticity, and softening hyperelasticity. A comparison of the three damage modelling techniques is summarised in Table 1.

### 3.1. Continuum Damage Mechanics (CDM)

In this section, various damage models based on continuum damage mechanics are reviewed chronologically, and all the reviewed CDM based damage models are summarized in Table 2. 

Blanco et al. [64] have developed a continuum damage model for soft tissues by including damage in fibre and matrix. The model was aimed to define the relationship between the mesoscopic structural mechanisms and macroscopic material parameters that dominate the inelastic behaviour in the soft fibrous tissues. The strain energy function, including the damage, is defined with the help of Neo-Hookean for the ground matrix and the Holzapfel Gasser Ogden (HGO) model for collagen fibres [24]. The strain energy function is defined as:

(15) $$\Psi =\frac{1}{2}\Psi_{vol}+\left(1-d_{dev}\right)\Psi_{dev}+\left(1-d_{f1}\right)\Psi_{af1}+\left(1-d_{f2}\right)\Psi_{af2}$$

And $\left\{\left(1-d_{dev}\;\right),\left(1-d_{af1}\;\right),\left(1-d_{af2}\;\right)\right\}$ are the reduction factors that introduce the inelastic material damage that occurs in the matrix and every set of fibres, complying with $0\leq d_{\alpha }\leq 1$ for $\alpha =\left\{dev,af1,af2\right\}$. The damage parameters are expressed as:

(16) $$d_{\alpha }=1-\frac{q_{\alpha }\left(r_{\alpha }\right)}{r_{\alpha }},\;\alpha =\left\{dev,af1,\;af2\right\}$$

Here $q_{\alpha }$ is defined as internal stress such as a variable whose variation defines the softening effect and damage threshold in the current configuration is defined by stress such as a variable, $r_{\alpha }$ satisfying $r_{\alpha }\leq r_{\alpha }^{0}$, with $r_{\alpha }^{0}$ the initial damage threshold.

The equivalent strain criterion of Simo and Ju [54] was used to define the energy norm of strain tensor as, $\tau :=\sqrt{2\Psi_{\alpha }}$ (i.e., the equivalent strain). The internal variable $r_{\alpha }$ can be integrated over time in the closed interval as:

(17) $$r_{\alpha }\left(t\right)=\begin{array}{c} \max\\ T\in \left[0,t\right] \end{array}\left[r_{\alpha }^{0},\sqrt{2\Psi_{\alpha }}\;\right]$$

In the equation above, T is time. The group of damage initiation criteria at any point of loading is given as:

(18) $$\sqrt{2\Psi_{\alpha }}-r_{\alpha }\leq 0$$

$q_{\alpha }$ is defined using the following hardening rule:

(19) $$\dot{q_{\alpha }}=H_{\alpha }\left(r_{\alpha }\right)\dot{r_{\alpha }},\;H_{\alpha }\left(r_{\alpha }\right)=\frac{\partial q_{\alpha }}{\partial r_{\alpha }},\;q_{\alpha }^{0}=r_{\alpha }^{0}$$

where $H_{\alpha }$ is the hardening/softening modulus given as:

(20) $$H_{\alpha }\left(q_{\alpha }\left(t\right),h\right)=-\frac{{\left(q_{\alpha }^{0}\right)}^{2-\chi }}{\left(2-\chi \right)}\frac{1}{G_{\alpha }^{f}}q_{\alpha }^{\chi }\left(t\right)h$$

Here χ is a parameter that defines the rate of softening, material parameter $G_{\alpha }^{f}$ specifies the surface density of dissipated energy, and h is the finite element characteristic size, which makes the model mesh dependent.

**Figure 5:**
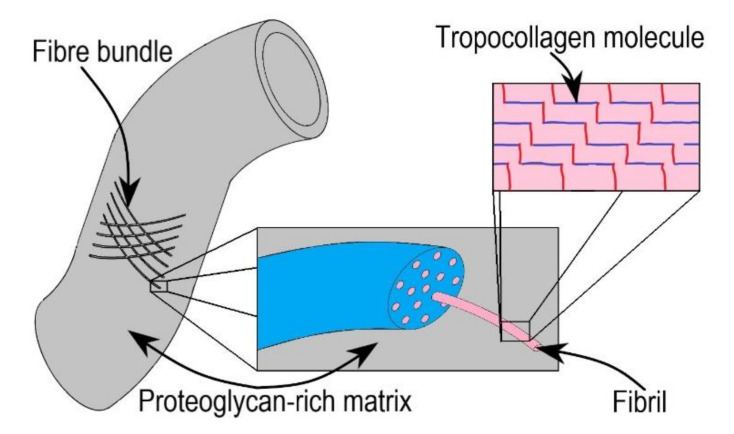
The hierarchical soft tissue model considered by Blanco et al. [64], Reprinted with permission from ref. [64], Copyright 2015 Elsevier.

Blanco et al. [64] derived the macroscopic damage model for soft tissues using mesoscopic parameters. These macroscopic parameters include the constitutive model parameters of the collagen fibres, namely $k_{1}$ and $k_{2}$, and inelastic parameters such as elastic stress threshold $\sigma_{f}^{u}$ and surface density of dissipated energy ${𝒤}_{f}^{u}$, wherein the elastic stretch threshold were defined using wavy fibril nature into consideration. The geometrical properties of mesoscopic fibril are modelled as staggered arrays of tropocollagen molecules using a two-dimensional Hodge–Petruska model. The mechanical characteristics of each fibril constituent were established by identifying modes of failures and their associated weak planes. The macroscopic behaviour was obtained by homogenizing the obtained properties of fibrils and proteoglycan rich matrix. The hierarchical soft tissue model considered by Blanco et al. [64] is shown in Figure 5.

Comellas et al. [65] developed a generalized damage model for quasi-incompressible hyperelasticity in a total Lagrangian finite-strain framework. The deviatoric part of the hyperelastic constitutive model was incorporated with the Kachanov-like reduction factor (1–d), which defines the softening effects. The damage model was implemented on Neo-Hookean and Ogden hyperelastic models. It adopts additively decomposed volumetric and deviatoric parts of Helmholtz free energy.

(21) $$\Psi \left(C,D\right)=\Psi_{vol}\left(J\right)+\left(1-d\right)\Psi_{dev}\left(\bar{C}\right)$$

The finite-strain based Kachanov effective stress using second Piola-Kirchhoff stress tensor (S) is given as:

(22) $$S=S_{vol}+\left(1-d\right){\bar{S}}_{dev}\;\mathrm{with}\;S_{vol}=-pJC^{-1}\;\mathrm{and}\;{\bar{S}}_{dev}=2\frac{\partial \Psi_{dev}}{\partial C}$$

Here p is the hydrostatic pressure given by $p=\frac{\partial \Psi_{\mathrm{vol}}}{\partial J}$.

The damaged surface $F=G\left(\tau \right)-G\left(\tau^{max}\right)=0$ determine the limits of the initiating point of non-linear behaviours. This model allows the usage of different energy-based norms. The damage evolution law $G\left(\tau \right)$ is defined in terms of the norm, and $G\left(\tau^{max}\right)$ is a scalar function where $\tau^{max}$ is the damage threshold. The criterion of Simo and Ju [54] was used to define the strain energy norm as $\tau =\sqrt{2\Psi_{dev}}$. They considered two types of damage evolution laws: (1) linear and (2) exponential forms. The damage variable d is expressed:

for linear softening,

(23) $$d=G\left(\tau \right)=\frac{1-\frac{S_{0}^{d}}{\tau }}{1+H}\;$$

for exponential softening,

(24) $$d=G\left(\tau \right)=1-\frac{S_{0}^{d}}{\tau }\exp\left[A\left(1-\frac{\tau }{S_{0}^{d}}\right)\right]$$

where

(25) $$\;H=\frac{-{\left(S_{dev}^{d}\right)}^{2}}{2g_{f}^{d}},\;A={\left[\frac{g_{f}^{d}}{{\left(S_{0}^{d}\right)}^{2}}-\frac{1}{2}\right]}^{-1}\;$$

Here  H and A are the dissipation parameters for linear and exponential softening, respectively. $S_{0}^{d}$ is the basic damage threshold stress and $g_{f}^{d}$ represents rupture energy per unit volume.

The energy norm differentiation of the evolution law is essential for evaluating the constitutive tangent tensor; the differentials are given as:

for linear softening,

(26) $$\frac{\partial G\left(\tau \right)}{\partial \tau }=\frac{-S_{0}^{d}}{\tau^{2}\left(1+H\right)}$$

for exponential softening,

(27) $$\frac{\partial G\left(\tau \right)}{\partial \tau }=\frac{S_{0}^{d}+A\tau }{\tau^{2}}\exp\left[A\left(1-\frac{\tau }{S_{0}^{d}}\right)\right]$$

Using the decoupled Helmholtz free energy Equation (21) along with Equation (27) with $d=G\left(\tau \right)$, the damage incorporated and additively decomposed material elastic tangent constitutive are given as:

(28) $${\mathbb{C}}^{tan}={\mathbb{C}}_{vol}^{tan}+{\mathbb{C}}_{dev}^{tan}\;\;with\;\left\{\begin{array}{c} {\mathbb{C}}_{vol}^{tan}=2\frac{\partial S_{vol}}{\partial C}=2p\frac{\partial \left(JC^{-1}\right)}{\partial C}+2JC^{-1}⊗p\frac{\partial p}{\partial C}\\ {\mathbb{C}}_{dev}^{tan}=2\frac{\partial }{\partial C}\left[\left(1-d\right){\bar{S}}_{dev}\right]=\left(1-d\right){\mathbb{C}}_{0dev}^{tan}-\frac{\partial G\left(\tau \right)}{\partial \tau }{\bar{S}}_{dev}⊗{\bar{S}}_{dev} \end{array}\right.$$

where ${\tilde{\mathbb{C}}}_{0dev}^{tan}=\left(2,\partial ,S_{\_dev}^{\circ }\right)/\partial C$ is deviatoric part of the damage free hyperelastic model, and $\partial G\left(\tau \right)/\partial \tau$ is damage dissipation defined in Equations (26) and (27) for linear and exponential softening, respectively. The damage parameters are defined using Matlab curve-fitting on experimental data of Martins et al. [72]. The quasi-incompressible volumetric part of the tangent modulus is unaffected by the damage, while the deviatoric part is influenced by the induced damage [65]. This model was applied for the rectus sheath to reproduce the experimental results numerically using their in-house finite element code (PLCd).

Polindara et al. [66] developed a damage model in line with Waffenschmidt et al. [73] to simulate balloon angioplasty in residually stressed blood vessels [66,74]. The model incorporates an anisotropic hyperelastic constitutive model defined by HGO [25] in the strain energy function for inclusion of the damage is given as:

(29) $$\Psi =\Psi_{vol}+\Psi_{dev}+f_{d}\left(\chi \right)\left(\Psi_{df1}+\Psi_{df2}\right)$$

To account for the fibre softening, a simple exponential damage function was introduced and is given by:

(30) $$f_{d}=\exp\left(\eta_{d}\left[\chi_{d}-\chi \right]\right)$$

where the evolution of local damage is controlled by the rate of change of damage variable χ, $\chi_{d}$ is the damage threshold that initiates the evolution, and $\eta_{d}$ is an exponential saturation parameter that controls the rate of damage evolution. Polindara et al. [66] have assumed damage parameters based on the stiffness degressive behaviour found in the inflation test experiments by Holzapfel [75]. Using an 8-noded hexahedral Q1Q1P0 element in Abaqus, they implemented the above damage model through UEL for non-local gradient-enhancement. Further, Polindara et al. [66] have used the same methodology and extended it to incompressible material with three damage variables [74]. These damage variables evolve independently from each other, accounting for damage in the matrix and the two fibre families.

Ferreira et al. [67] provided a general framework for inducing damage in hyperelastic materials. The computational framework to locally model the anisotropic damage is considered in the non-linear geometry. This model assumes that the stretch patterns in the soft tissues result in pathological conditions. Further, they cause the stable degradation of the collagen fibres and the ground matrix of the soft tissue. The fully anisotropic hyperelastic material in the form of the strain energy density was defined as

(31) $$\Psi =\Psi_{vol}\left(J\right)+\left(1-d_{dev}\right)\Psi_{dev}\left(\bar{F}\right)+\left(1-d_{ani}\right)\Psi_{ani}+\Phi_{dev}\left(d_{dev}\right)+\Phi_{ani}\left(d_{ani}\right)$$

where $d_{dev},{\;d}_{ani}\in \left[0,\;1\right]$, are the internal damage variables for matrix and fibres, and functions $\Phi_{dev}$ and $\Phi_{ani}$ represent the damage propagation in the material before the tear propagation in matrix and fibres, respectively.

The proposed model adopts the Cauchy stress and effective tangent moduli tensor by an additive composition of each contribution.

(32) $$\sigma =\sigma_{vol}+\left(1-d_{dev}\right)\sigma_{dev}+\left(1-d_{ani}\right)\sigma_{ani}$$

(33) $$c=c_{jr}+c_{vol}+c_{dev}+c_{ani}$$

The parameters d for the damage evolution for matrix and fibres are represented by an irreversible equation. The damage parameters defined in terms of the reduction factor is given as:

(34) $$d=1-\bar{g}$$

The reduction factor is obtained in terms of the equivalent strain $\Xi_{s}$ at time s as defined by Simo [68]. The maximum value evolved during the deformation history till the current time is used for the evaluation of the reduction factor $\bar{g}$.

(35) $$\Xi_{t}=\begin{array}{c} max\\ s\in \left(0,t\right) \end{array}\sqrt{2\bar{U}}$$

In material degradation, the law is given by:

(36) $$\bar{g}=\left\{\begin{array}{c} 1,\;\;\;\;\;\;\;\;\;\;\;\;\;\;\;\;\;\;\Xi_{t}<\Xi_{min}\\ \frac{1-\exp\left(\beta \left(\Xi_{t}-\Xi_{\max}\right)\right)}{1-\exp\left(\beta \left(\Xi_{\min}-\Xi_{t}\right)\right)},\;\Xi_{\min}<\Xi_{t}<\Xi_{max}\\ 0,\;\;\;\;\;\;\;\;\;\;\;\;\;\;\;\;\;\;\;\Xi_{t}>\Xi_{max} \end{array}\right.$$

The thresholds $\Xi_{\min}$ and $\Xi_{max}$ are defined based on the tensile experiments. In the above equation, β is the averaging operator that takes a bell-shaped form and is expressed as:

(37) $$\beta \left(x,\xi \right)=\frac{1}{\Omega \left(x\right)}{\left(1-\frac{{\left(x-\xi \right)}^{2}}{l_{r}^{2}}\right)}^{2}$$

Here x is the point in the material, Ω is the defined finite volume containing a set of points ξ and $l_{r}$ is the characteristic length.

The developed damage formulation was tested for hyperelastic constitutive laws such as Neo-Hookean, Moony–Rivlin, Ogden, Humphrey–Martins, and HGO [67]. Wherein the damage parameters are set by admitting the degradation of matrix and fibres. The finite element simulations for internally pressurized healthy artery is implemented using various Abaqus user subroutines.

Rausch et al. [68] developed a soft tissue damage model by combining the continuum damage theory with smoothened particle hydrodynamics (SPH). The material response was assumed to be non-linear hyperelastic with a single family of fibres. The considered strain energy function for the fibre orientation was defined by HGO [25], as follows

(38) $$\Psi_{0}=\frac{\mu }{2}\left(I_{1}-3\right)-K\left(J-1\right)+\frac{k_{1}}{4k_{2}}\left[\exp\left(k_{2}{\left(I_{4}-1\right)}^{2}\right)-1\right]$$

The damage initiation was based on the formulation of Simo [76]. $\Psi_{0}$ is the strain energy free from damage $d\in \left[0,1\right]$. The concentration of d increases with the increase in the maximum principal stretch in the material beyond the critical stretch $\lambda_{crit}$.

(39) $$\Psi =\left(1-d\right)\Psi_{0}$$

An exponential evolution equation is considered for the damage function,

(40) $$d=\left\{\begin{array}{c} \exp\left({\left(\frac{\left(\lambda_{m}-\lambda_{crit}\right)}{\tau }\right)}^{2}\right)-1,\lambda_{m}\geq \lambda_{crit}\\ 0,\;\lambda_{m}<\lambda_{crit} \end{array}\right.$$

The maximum principal stretch ($\lambda_{m}$) from the previous increment is evaluated as $\lambda_{m}\left(t\right)=\underset{s\in \left[-\infty ,t\right]}{\max}\lambda \left(s\right)$ at a rate determined through τ. Damage in the material initiates once $\lambda_{m}$ exceeds $\lambda_{crit}$ and it is irreversible. These damage parameters are defined from sensitivity analysis of damage parameters on material behaviour. The study of Rausch et al. [68] is aimed to explore the applicability of the Normalized Total Lagrangian-SPH method in soft tissue mechanics. The Rausch et al. [68] damage model was used to simulate material discontinuities in a single edge notch soft tissue specimen under uniaxial tension. The SPH simulations are carried using MATLAB, and for validating the SPH results, similar FEM simulations are carried using open-source FEbio.

Fathi et al. [69] had implemented a non-local integral-damage model to overcome the numerical artefacts such as mesh dependency and spurious localization in soft tissues. This damage model, which considers large deformation, was used to simulate aortic dissections. The strain energy equation, consisting of the damage variables, is given by:

(41) $$\Psi =K\ln J^{2}+\left(1-d_{m}\right)\left\{c_{1}\left(1-{\bar{I}}_{1}\right)+c_{2}\left(1-{\bar{I}}_{2}\right)\right\}\;\;\;\;\;\;\;\;\;\;\;\;\;\;\;\;\;+\left(1-d_{f}\right)\frac{k_{1}}{2k_{2}}\left\{\exp\left(k_{2}\kappa \langle {\bar{I}}_{1}+\left(1-3\kappa \right){\bar{I}}_{fi}-I_{0i}\rangle^{2}\right)-1\right\},\;i=1,2$$

where $c_{1}$, and $c_{2}$, are material parameters, and ${\bar{I}}_{f1}={\bar{I}}_{4}$ and ${\bar{I}}_{f2}={\bar{I}}_{6}$ are deviatoric invariants. The Macaulay bracket ($\langle ■\rangle =\left(\left|■\right|+■\right)\;/2$) assumes the fibres can support tension only. The state of folding fibres and their mobilization is defined using dimensionless parameter $I_{0i}$.

The damage function is defined as:

(42) $$\tilde{G}\left(\Xi_{t}^{k}\right)=1-d_{k}=\left\{\begin{array}{cc} 1 & if\;\Xi_{t}^{k}<\Xi_{min}^{k}\;\\ \frac{\Xi_{\max\;}^{k}-\Xi_{t}^{k}}{\Xi_{\max\;}^{k}-\Xi_{\min\;}^{k}}\exp\left[\beta_{k}\left(\frac{\Xi_{\max\;}^{k}-\Xi_{t}^{k}}{\Xi_{\max\;}^{k}-\Xi_{\min\;}^{k}}\right)\right] & if\;\Xi_{min}^{k}\leq \Xi_{t}^{k}\leq \Xi_{max}^{k}\\ 0 & if\;\Xi_{t}^{k}>\Xi_{max}^{k} \end{array}\right.$$

where the total and initial equivalent strain are defined using $\Xi_{max}^{k}$ and $\Xi_{min}^{k}$, respectively and the exponential coefficient $\beta_{k}\;$ represents the damage saturation. These damage parameters are defined based on Martins et al. [72] for the abdomen rectus sheath and Weiss et al. [77] for the ligament.

The strain evolution parameter is defined in similar lines with Simo and Ju [55].

(43) $$\Xi_{t}^{k}=\begin{array}{c} \max\;\\ s\in \left(-\infty ,t\right) \end{array}\sqrt{2\Psi_{0}^{k}\left(s\right)\;};\;\;\;\;k=m,f_{1},f_{2}$$

where the subscript 0 represents the intact material, and the superscript k represents the particular component of the strain energy, i.e., m, $f_{1}$ and $f_{2}$ corresponds to the matrix, and the first and second family of fibres, respectively.

The damage model proposed by Fathi et al. [69] was simulated with an integral-type non-local scheme that can be implemented on geometrically nonlinear soft tissue by overcoming mesh dependency. The role of the collagen fibres is also studied with the damage model and was found to be predominant. The mechanical response of the soft tissue varies with the position and the direction of the fibre. The same study also proposed soft tissue tear simulations with the combination of damage model and XFEM or meshless methods.

An anisotropic multi-physics damage model to capture damage initiation and propagation in annulus fibrosus was proposed by Gao et al. [70]. This model was derived based on the damage model proposed by Balzini et al. [37] by integrating continuum mixture theory and CDM. The constitutive strain energy equation is given by:

(44) $$\Psi_{\mathrm{AF}}=\Psi_{m}+\underset{f=4,6}{\sum }\Psi_{f}$$

with

(45) $$\Psi_{m}=\frac{\mu }{2}\left(I_{1}-3\right)-\mu \ln J+\lambda {\left(\phi_{0}^{w}\right)}^{2}\left[\frac{J-1}{\phi_{0}^{w}}-\ln\left(\frac{J-1}{\phi_{0}^{w}}+1\right)\right]$$

and

(46) $$\Psi_{f}=\frac{k_{1}}{2k_{2}}\left\{H\left(I_{f}-1\right)\exp\left[I_{f}-1\right]-1\right\},\;f=4,6$$

where the intact strain energy density function of the matrix is given by $\Psi_{m}$ and fibre is given by $\Psi_{f}$. $\phi_{0}^{w}$ represents the reference state water volume fraction, λ is material constants, $I_{1}$ is the first principal invariant of C, and H is the Heaviside step function.

After introducing the damage parameters, the strain energy function becomes:

(47) $$\Psi_{\mathrm{AF}}=\left(1-d_{m}\right)\Psi_{m}+\underset{f=4,\;6}{\sum }\left(1-d_{f}\right)\Psi_{f}$$

In the reported study [71], the damage in the matrix was neglected, and only the damage contribution of the fibre was solely considered. The damage evolution variable was defined as:

(48) $$d_{f}=d_{\max\;}\left[1-\exp\left(-\frac{\beta_{i}}{\gamma }\right)\right]$$

$d_{\max}$ and γ is the damage parameters and the internal damage variable $\beta_{i}$ defined in terms of function $\Psi_{f}$ as:

(49) $$\beta_{i}=\begin{array}{c} \max\;\\ 0\leq \tau \leq t \end{array}\Psi_{f}\left(\tau \right)-{\hat{\Psi }}_{f}$$

where ${\;\hat{\Psi }}_{f}$ is the limiting strain energy density of fibre beyond which damage occurs. Damage parameters are approximated from the experimental data of Pezowicz [78].

The study of Gao et al. [69] was aimed to develop a multi-physics damage model to study damage in annulus fibrosus. The continuum mixture theory was used to develop a coupled field problem that considers the water content along with the soft tissue material. While CDM is used to model the damage in the coupled field problem. Numerical studies of annulus fibrosis for damage under compression found the results are sensitive to ${\;\hat{\Psi }}_{f}$ then the other damage parameters.

A damage model to predict damage growth before the rupture in ascending thoracic aneurysms was reported by Mousavi et al. [71]. The proposed model is layer-specific and uses a constrained mixture theory that inherently considers internal/residual stresses. As per constrained mixture theory, distinct strain energy density was proposed for every individual constituent concerned with the contribution of its mass function. The damage was assumed to occur in fibre constituents, i.e., elastin and collagen, and the strain energy function was given as:

(50) $$\Psi =2\Psi_{vol}+\rho_{m}\Psi_{m}+\left(1-d_{e}\right)\rho_{e}2\Psi_{dev}+\overset{n}{\underset{i=1}{\sum }}\left(1-d_{ci}\right)\rho_{ci}\Psi_{ci}$$

where subscripts e, ci, and m represent elastin fibre, collagen fibres, and smooth muscle cells, respectively; $d_{j}$ are respective damage parameters and $\rho_{j}$ are specific mass fractions. The constitutive strain energy density in tension and compression for collagen and smooth muscle cells, distinctly defined as:

(51) $$\Psi_{ci}=\frac{k_{1}^{ci,t}}{2k_{2}^{ci,t}}\left\{\exp\left[k_{2}^{ci,t}{\left({\bar{I}}_{4}^{ci}-1\right)}^{2}\right]-1\right\},\;\mathrm{under}\;\mathrm{tension}$$

(52) $$\Psi_{ci}=\frac{k_{1}^{ci,c}}{2k_{2}^{ci,c}}\left\{\exp\left[k_{2}^{ci,c}{\left({\bar{I}}_{4}^{ci}-1\right)}^{2}\right]-1\right\},\;\mathrm{under}\;\mathrm{compression}$$

and

(53) $$\Psi_{m}=\frac{k_{1}^{m,t}}{2k_{2}^{m,t}}\left\{\exp\left[k_{2}^{m,t}{\left({\bar{I}}_{4}^{m}-1\right)}^{2}\right]-1\right\},\;\mathrm{under}\;\mathrm{tension}$$

(54) $$\Psi_{m}=\frac{k_{1}^{m,c}}{2k_{2}^{m,c}}\left\{\exp\left[k_{2}^{m,c}{\left({\bar{I}}_{4}^{m}-1\right)}^{2}\right]-1\right\},\;\mathrm{under}\;\mathrm{compression}$$

A linear softening law is used to evaluate the damage variable, as described by Comellas et al. [65]. The apparent density of damage is assumed to evolve with mechanical loading.

(55) $$d_{j}=G_{j}\left(\psi_{j}\right)=\frac{1-\frac{\psi_{j}^{0}}{\psi_{j}}}{1+H_{j}}$$

here

(56) $$H_{j}=-\frac{{\left(\psi_{j}^{0}\right)}^{2}}{2\omega_{j}},\;\omega_{j}=\frac{\Omega_{j}}{L_{o}}\;\mathrm{and}\;\psi_{j}=\sqrt{2\Psi_{j}}$$

where $j\in \left\{e,ci\right\}$, $\psi_{j}^{0}$ is the basic damage threshold and $\omega_{j}$ represents the rupture energy per unit volume that is defined based on the maximum fracture energy dissipated per unit area, $\Omega_{j}$, and $L_{o}$ characteristic element length. Buldge inflation experiments were performed, and with the help of curve fitting to experimental data, damage parameters are defined. Numerical simulations of the developed damage model were carried in a commercial finite element software (Abaqus) using a user-defined subroutine (UMAT). Human aortic aneurysm specimens were also simulated under uniaxial tension, patient-specific over-pressurization, and bulge inflation tests.

A damage model to accurately capture the phenomena of passive damage in arteries was proposed by Ghasemi et al. [35]. These passive damage phenomena include Mullins effects, hysteresis, permanent set, and the effect is captured up to the level of rupture. In the model of Ghasemi et al. [35], the damage is assumed to take place in elastic fibre and collagen fibre only. The constitutive equation with damage variables incorporated is defined as:

(57) $$\Psi =\Psi_{vol}+\Psi_{dev}+\underset{M_{ef}=M_{4ef},M_{6ef}}{\sum }\frac{k_{1}^{ef}}{2k_{2}^{ef}}\left\{\exp\left[k_{2}^{ef}\left(1-d^{ef}\right){\left({\bar{I}}_{M_{ef}}-1\right)}^{2}\right]-1\right\}\;\;\;\;\;\;\;\;\;\;\;\;+\underset{M_{cf}=M_{4cf},M_{6cf}}{\sum }\frac{k_{1}^{cf}}{2k_{2}^{cf}}\left\{\exp\left[k_{2}^{cf}\left(1-d^{cf}\right){\left(\kappa {\bar{I}}_{1}+\left(1-3\kappa \right){\bar{I}}_{M_{cf}}-1\right)}^{2}\right]-1\right\}$$

where material parameters $k_{1}^{ef}$& $k_{2}^{ef}$ and $k_{1}^{cf}\&\;k_{2}^{cf}$ corresponds to elastin fibre and collagen fibre, respectively. The deviatoric invariants ${\bar{I}}_{M_{ef}}$ and ${\bar{I}}_{M_{cf}}$ represent the square of stretch in elastin fibre and collagen fibre, respectively. The damage of elastin and collagen fibres are represented by $d^{ef}$ and $d^{cf}$, respectively.

The damage functions capture the continuous and discontinuous softening using two internal variables $\beta_{i}$ and $\gamma_{i}$ respectively, and defined as:

(58) $$\beta_{i}=\langle {\tilde{\beta }}_{i}-{\tilde{\beta }}_{i}^{ini}\rangle$$

Here i=ef for elastin fibres and i=cf for collagen fibres, ${\tilde{\beta }}_{i}^{ini}$ the initial parameter that ensures the damage evolution. Macaulay brackets, ($\langle ■\rangle =\left(\left|■\right|+■\right)\;/2$) ensures only positive values. ${\tilde{\beta }}_{i}$ is constructed as per the changes in the pseudo-invariant over the complete loading history, as:

(59) $${\tilde{\beta }}_{i}=\int_{0}^{t}\langle {\bar{I}}_{M_{i}}^{*}\rangle ds$$

where $\gamma_{i}$ is defined based on the maximum value of ${\bar{I}}_{M_{i}}^{*}$ evolved during the loading history till the current loading state, and it is given as:

(60) γi=I¯S∈0, Smax〈I¯Mi*−I¯Mi*ini〉

The damage variable is constructed as:

(61) $$d^{i}=d_{\infty }^{i}\left[1-\exp\left(-\frac{\gamma_{i}}{\gamma_{\infty_{i}}}\right)\right]\left[1-\exp\left(-\frac{\beta_{i}}{\beta_{S_{i}}}\right)\right],\;d^{i}\in \left[0,1\right]$$

where the predefined variable $d_{\infty }^{i}$ limits the overall damage in elastin fibres and collagen fibres, while the parameters $\beta_{S_{i}}$ and $\gamma_{\infty_{i}}$ represent the continuous and discontinuous softening of the soft tissues, respectively. Ghasemi et al. [35] have used an inverse FE algorithm to draw damage parameters using experimental results of uniaxial tension tests.

An anisotropic microsphere-based approach to model the damage in soft vascular tissue was developed by Saez et al. [79]. A microsphere-based damage approach for modelling damage by considering the microstructure is initially developed by Miehe et al. [80] and Dal and Kaliske [81] for rubbers. Saez et al. [79] have extended the model for anisotropic soft tissues by neglecting fibre crosslinks and sliding between fibres and the surrounding matrix. However, the fit between the experimental stress-stretch data and that predicted by the microstructural model of Saez et al. [79] was reported to be not satisfactory by Pena et al. [82]. Particularly, the correlation between the experimental data and the prediction by the microstructural damage model was found to be worse, as compared to the phenomenological model by Pena [83]. Hence detailed discussion on the microstructural damage model by Saez et al. [79] is not included in this review.

### 3.2. Pseudo-Elasticity

In 2015, Pierce et al. [84] had modelled the material response of human thoracic and abdominal aortic tissues using the damage model proposed by Weisbecker et al. [41]. Pierce et al. damage model differentiated between physiological and supraphysiological loading. They compared the damage response in healthy and diseased vascular tissues using it. In particular, for the diseased tissues, abnormal aortic aneurysms were considered (abnormal swelling or bulge in the wall of the artery is known as an aortic aneurysm). The elastic damage model postulated in terms of the strain energy density is given as:

(62) $$\Psi =\Psi_{vol}+\Psi_{dev}+\underset{i=4,6}{\sum }\left[\eta_{fi}\left(\Psi_{dfi}\right)+\Phi_{fi}\left(\eta_{fi}\right)\right]$$

where $\Phi_{fi}$ is the smooth damage function of the fibres, and $\eta_{fi}\in \left[0,\;1\right]$ represents the damage variables of the two fibre families given as:

(63) $$\eta_{fi}=1-\frac{1}{r_{f}}\mathrm{erf}\left[\frac{1}{m_{f}}\left(\Psi_{dfi}^{max}-\Psi_{dfi}\right)\right]$$

where $\Psi_{dfi}^{max}$ is the maximum strain energy evolved during the deformation history, $r_{f}>1$ defines the maximum allowable damage in the fibres subjected to loading, and $m_{f}>0$ defines the accumulation of softening in the fibres. The minimum of the damage variable characterizes the damage induced in the fibres as:

(64) $$\eta_{fi}^{min}=1-\frac{1}{r_{f}}\mathrm{erf}\left(\frac{1}{m_{f}}\Psi_{dfi}^{max}\right)$$

The damage model presented includes the effect of damage on fibres only, and the damage parameters are defined using curve-fitting on uniaxial tension experimental data. Pierce et al. [84] study involve constitutive modelling for damage, damage experiments, and statistical data analysis to identify the material and damage parameters. A similar pseudo-elasticity model is used by He et al. [85] to simulate damage of the artery during stent deployment.

Holzapfel and Ogden [86] have proposed a progressive damage model for the collagen fibres based on the pseudo-elastic model. Their model considers both the Mullins effect and cross-linking between the collagen fibres. The same model is validated for an experimental behaviour of rat tail tendon fibres. The damage induced strain energy density is defined as:

(65) $$\Psi =\Psi_{dev}+\eta \Psi_{af1}+\phi \left(\eta \right)$$

where η is a dimensionless damage variable that introduces the damaging effect and $\phi \left(\eta \right)$ is some damage measure. The damage variable and damage function are given by

(66) $$\eta =\exp\left(-\frac{\Psi_{af1}-\Psi_{af1}^{c}}{m}\right)$$

(67) $$\phi \left(\eta \right)=m\eta \log\eta +\left(1-\eta \right)\left[m+\Psi_{af1}^{c}\right]$$

Here parameter m>0 has the same dimension as Ψ and $I_{4c}=\lambda_{c}^{2}$ is the critical stretch value of $I_{4}$, which is responsible for the initiation of the damage, i.e., η decreases from 1 for the stretch $\lambda >\lambda_{c}$ down to $\eta_{f}$ for a failure occurs at $\lambda =\lambda_{f}$.

The collagen fibres cross-links are included with the unit vectors $L^{+}$ and $L^{-}$ around the collagen fibre direction $a_{1}$. The unit vectors $L^{+}$ and $L^{-}$ represents the fibre cross-links are logically symmetric about $a_{1},$ and the operation of the deformation gradient F on them given as:

(68) $$L^{\pm }=\pm c_{0}a_{1}+s_{0}a_{2}$$

(69) $$FL^{\pm }=\pm c_{0}Fa_{1}+s_{0}Fa_{2}$$

**Figure 6:**
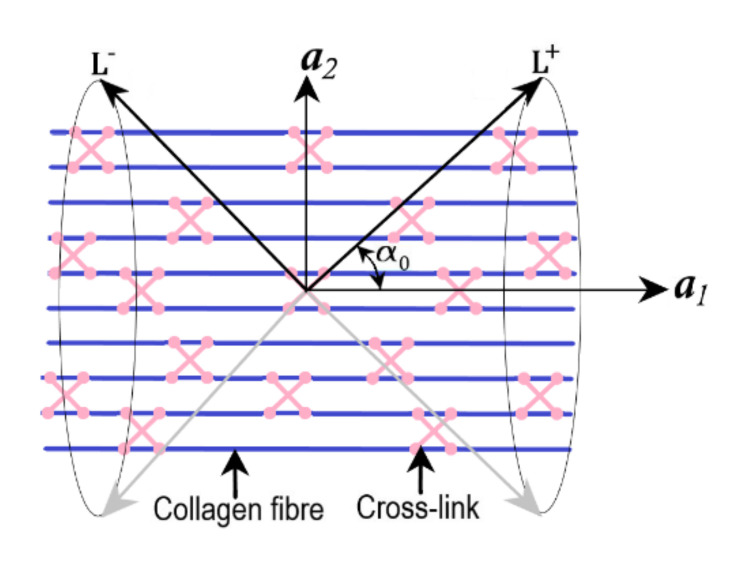
Collagen fibre with the cross-links structure proposed by Holzapfel and Ogden [87] (Reprinted from ref. [88]).

For conciseness, the representation of $s_{0}=\sin\alpha_{0}$ and $c_{0}=\cos\alpha_{0}$ were used by Holzapfel and Ogden [86], where $\alpha_{0}$ defines the relative orientation of fibre cross-link vectors ($L^{+}$ and $L^{-}$) with reference to the direction of $a_{1}$ (Figure 6). To model the effect of the collagen cross-links, Holzapfel and Ogden [86] introduced a couple of pseudo invariants $I^{\pm }$ and $I_{8}^{\pm }$. Wherein $I^{\pm }$ represents the squares of the stretches in the cross-link directions and $I_{8}^{\pm }$ describes the coupling between the collagen fibre and cross-links.

(70) $$I^{\pm }=c_{0}^{2}I_{4}\pm 2s_{0}c_{0}\left(Ca_{1}\right)\cdot a_{2}+s_{0}\left(Ca_{2}\right)\cdot a_{2}$$

(71) $$I_{8}^{\pm }=\pm c_{0}I_{4}+s_{0}\left(Ca_{1}\right)\cdot a_{2}$$

where C is the right Cauchy-Green tensor.

For uniaxial tension where the stretch λ in the fibre direction $a_{1}$ gives $Fa_{1}=\lambda a_{1}$ and $Fa_{2}=\lambda^{-\frac{1}{2}}a_{2}$. The deformation gradient acting on the cross-link vectors gives,

(72) $$FL^{\pm }=\pm c_{0}\lambda a_{1}+s_{0}\lambda^{-\frac{1}{2}}a_{2}$$

and additionally, gives the cross-link directions and quantities as

(73) $$I\equiv I^{\pm }=c_{0}^{2}\lambda^{2}+s_{0}^{2}\lambda^{-1},\;I_{8}=I_{8}^{+}=c_{0}\lambda^{2}\;\mathrm{and}\;I_{8}^{-}=-I_{8}^{+}$$

The specific strain energy function with isotropic strain energy, anisotropic strain energy along with the quadratic terms correlating the cross-links and fibre/cross-link density interactions given as:

(74) $$\Psi =\Psi_{dev}+\eta \Psi_{af1}^{c}+\frac{1}{2}\nu {\left(I-1\right)}^{2}+\frac{1}{2}\kappa {\left(I_{8}-c_{0}\right)}^{2}$$

where the stress-like parameters ν and κ correlate the cross-links and interactions, respectively. Wherein ν represents the density of cross-links, and κ represents the measure of the interaction energy. For instance, the damage variable for uniaxial tension is given by:

(75) $$\eta =\exp\left[-\frac{k_{1}}{2mk_{2}}\left\{\exp\left[k_{2}{\left(\lambda -1\right)}^{2}\right]-\exp\left[k_{2}{\left(\lambda_{c}-1\right)}^{2}\right]\right\}\right]$$

The Cauchy stress σ becomes:

(76) $$\sigma =\mu \left(\lambda^{2}-\lambda^{-1}\right)+2k_{1}\eta \lambda^{2}\left(\lambda^{2}-1\right)\exp\left[k_{2}{\left(\lambda^{2}-1\right)}^{2}\right]+4\nu \left(I-1\right)\left(c_{0}^{2}\lambda^{2}-s_{0}^{2}\lambda^{-1}\right)\;+4\kappa \left(I_{8}-c_{0}\right)c_{0}\lambda^{2}$$

The same model was applied for planar deformations for the case of simple shear, wherein both collagen fibres and cross-links are assumed to be lying in a plane [86,87]. Wherein the critical stretch (damage parameter) is defined using least square curve-fitting on uniaxial tension experimental data. The proposed model focuses on damage at the collagen fibre level and does not consider the fibrils and proteoglycans structure.

### 3.3. Hyperelastic Softening

An invariant-based constitutive model that accounts for damage for skin was developed by Li and Luo [63]. The skin was assumed to have two symmetric families of fibre, and their structures were constant across its depth. Their damage model was developed based on the HGO type strain energy function and the Volokh damage model [89,90]. The strain energy function that incorporates the damage is given by:

(77) $$\Psi =\frac{\mu }{2}\left[\left(I_{1}-3\right)-\frac{{\left(I_{1}-3\right)}^{m+1}}{\left(m+1\right){\left(\zeta -3\right)}^{m}}\right]+\frac{k_{1}}{k_{2}}\left\{\exp\left(k_{2}A^{2}\right)-1-\frac{2k_{2}A^{n+2}}{\left(n+2\right){\left(\xi^{2}-1\right)}^{n}}\right\}$$

where $A=\lambda_{f}^{2}-1$, with $\lambda_{f}=\sqrt{\kappa I_{1}+\left(1-3\kappa \right)I_{4}}$ is the fibre stretch, and m, n, ζ, and ξ are phenomenological damage parameters to induce softening. In particular, when damage occurs, m defines the stretch curve sharpness, and ζ represents the value of $I_{1}$ associated with the matrix. n is the equivalent contrary to m, and ξ represents the onset of damage of the fibre in terms of $\lambda_{f}$.

The softening hyperelasticity model is extended to human artery adventitia, where failure is considered as part of the constitutive model [88]. This model is developed using the HGO material model [34] and the energy limiters. The limiting value for the strain energy and failure energy is enforced using the energy limiters that restrict the stresses in the constitutive equations [90]. Volokh [88] proposed the strain energy function for adventitia as

(78) $$\psi =\frac{\mu }{2}\;\left(I_{1}-3\right)H\left(\zeta_{4}\right)H\left(\zeta_{6}\right)+H\left(\zeta_{4}\right)\left\{\psi_{4}^{f}-H\left(\zeta_{4}\right)\psi_{4}^{e}\right\}+H\left(\zeta_{6}\right)\left\{\psi_{6}^{f}-H\left(\zeta_{6}\right)\psi_{6}^{e}\right\}$$

where the step function $H\left(\zeta_{i}\right)$ is defined as $H\left(\zeta_{i}\right)=0$ for $\zeta_{i}<0$, else $H\left(\zeta_{i}\right)=1$, and strain energies $\psi_{i}^{f}$ & $\psi_{i}^{e}$ of collagen fibres are defined as:

(79) $$\psi_{i}^{f}=\Phi_{i}m_{i}^{-1}\Gamma \left(m_{i}^{-1},\;0\right),\;\psi_{i}^{e}=\Phi_{i}m_{i}^{-1}\Gamma \left(m_{i}^{-1},\;W_{i}^{m_{i}}\Phi_{i}^{-m_{i}}\right),\;i=4,6$$

Here the gamma function is defined as $\Gamma \left(s,x\right)=\int_{x}^{\infty }a^{s-1}\exp\left(-a\right)da$, and $\Phi_{i}$, $m_{i}$ material failure parameters. In particular, $\Phi_{i}$ is the energy limiter that describes the average bond energy. The strain energy function $W_{i}$ for the aligned intact fibres is given as,

(80) $$W_{i}=\Psi_{afi}=\frac{k_{1}}{2k_{2}}\left[\exp k_{2}{\left(I_{i}-1\right)}^{2}-1\right]\;,\;i=4,6$$

In this model, fibres contribute to strain energy only in tension, i.e., $I_{4}>1$ and $I_{6}>1$.

In Equation (78), $\zeta_{i}\in \left(-\infty ,\;0\right]$ is a switch parameter, and its evolution is defined as:

(81) $$\dot{\zeta_{i}}=-H\left(\epsilon_{i}-\frac{\psi_{i}^{e}}{\psi_{i}^{f}}\right),\;\zeta_{i}\left(t=0\right)=0,\;i=4,\;6$$

where $\epsilon_{i}$ is a dimensionless precision constant that is defined as $0<\epsilon_{i}\ll 1$.

In the proposed model, the material response is hyperelastic when $\psi_{i}^{e}<\psi_{i}^{f}$. The strain energy remains constant ($W_{f}=\psi_{i}^{f})$ and prevents healing in the material & enables energy dissipation. The switch parameters differentiate the elastic and damage response, i.e., $\zeta_{i}=0$ for elastic response and $\zeta_{i}<0$ for irreversible damage, and strain is dissipated. The step multipliers assume that the damage in either family of fibres results in whole tissue failure. Volokh has defined the damage parameters using least square curve-fitting on uniaxial tension experimental results [88].

## 4. Rupture Modelling

Propagation of crack in soft tissues is considered as tear propagation/rupture. Soft biological tissue exhibits a complex rupture phenomenon due to the presence of collagen fibres. These collagen fibres in the soft tissue make them resistant to defects [30]. Crimped collagen fibres in the vicinity of the crack tip gradually straighten, providing resistance to crack propagation [16,31]. A tear propagation approach should be capable of capturing such complex phenomena. Various rupture modelling approaches used for soft tissues are reviewed in the present section.

The above-discussed damage approaches operate within the standard continuum mechanics approach wherein the displacement fields are continuous. In contrast, soft tissue rupture is macroscopic damage, which is considered as the point where the crack initiates, i.e., where discontinuity in the material occurs. Such a macroscopic damage phenomenon is dealt with by the fracture mechanics approach [55,91]. Fracture mechanics (FM) involves discontinuous displacement fields and uses techniques such as extended finite element method (XFEM), cohesive zone modelling (CZM), crack phase-field modelling (CPFM), etc. In rupture modelling, XFEM and CZM are the numerical techniques to simulate fracture, while CPFM is a mathematical model. The initiation and propagation criteria need to be defined for modelling the propagating crack. The modelling of the crack propagation in the conventional finite element method (FEM) requires re-meshing, which is a cumbersome task.

In contrast, XFEM can model discontinuities and their propagation by overcoming the problem of re-meshing [92]. XFEM is an extension of FEM, which is based on the concept of partition of unity that introduces the enrichment functions associated with additional degrees of freedom [93]. The Heaviside functions and asymptotic crack-tip fields represent the enriched discontinuous displacement fields and crack-tip singularity [94]. The XFEM has been extensively used for surgical cutting simulations [95,96,97], rupture simulations of soft-hydrated tissues [98,99], and arterial dissections [100,101].

**Figure 7:**
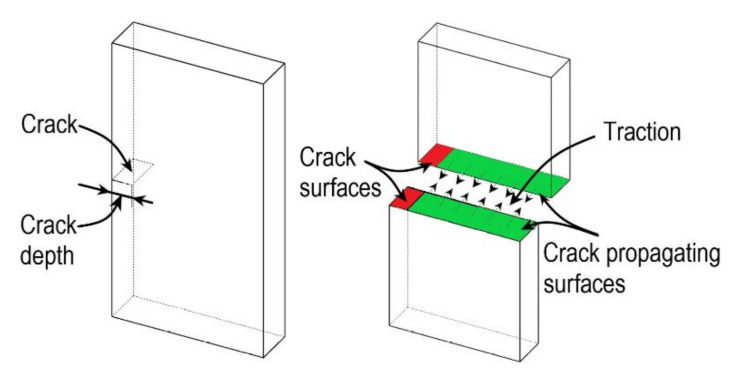
Cracked object.

In CZM, a cohesive surface is placed in the intact region of the material where the crack propagates, as shown in Figure 7. The cohesive surface is modelled with special elements called cohesive elements. The crack propagation is modelled with the help of traction separation law, i.e., when the opening displacement reaches a limiting value, the traction along the surface disappears [102,103]. Pandolfi and co-workers have extensively applied CZM on arteries, also proposed an anisotropic extension to irreversible cohesive law [104,105,106]. Gasser and Holzapfel [107] have simulated arterial strip dissection by adopting the modality of combined CZM, and XFEM approaches introduced by Moes and Belytchko [108].

In contrast to XFEM and CZM, CPFM models discontinuity as a separate continuous field, and it is coupled with the deformation field to model the crack propagation. The minimum energy variational principle is used for numerical analysis to solve the coupled field. In line with XFEM, a damage initiation criterion is needed for the crack propagation, i.e., the crack phase-field evolution initiates. Miehe and co-workers made a phenomenal development of CPFM with the thermodynamically consistent and algorithmically robust formulation [109,110]. Gultekin introduced the application of CPFM in biomechanics, which was later used for simulating aortic dissections [48,111,112,113]. Raina and Miehe [114] proposed an anisotropic failure criterion for computing driving forces during damage growth in soft biological tissues. Furthermore, numerical aspects associated with aortic dissections were investigated in the studies by Raina and Miehe [114] and Gultekin et al. [48,112].

### 4.1. Extended Finite Element Method (XFEM)

In conventional FEM, the displacement field is interpolated using shape functions ($N^{I}$) and nodal degrees of freedom (${\underline{u}}_{c}^{I}$). Additionally, in XFEM, to model the crack and its propagation, the displacement field is incorporated with Heaviside function (H) and enriched degrees of freedom (${\underline{u}}_{e}^{I}$) as follows,

(82) $$\underline{u}=\overset{n_{el}}{\underset{I=1}{\sum }}N^{I}{\underline{u}}_{c}^{I}+H\overset{n_{el}}{\underset{I=1}{\sum }}N^{I}{\underline{u}}_{e}^{I}+\overset{n_{el}}{\underset{I=1}{\sum }}\overset{4}{\underset{\alpha =1}{\sum }}F_{\alpha }N^{I}{\underline{u}}_{\alpha }^{I}$$

where the index (I) runs from 1 to $n_{el}$ (number of nodes per element). $F_{\alpha }$ asymptotic crack tip functions and ${\underline{u}}_{\alpha }^{I}$ nodal enriched degrees of freedom. The third term in the displacement characterizes the stress singularity at the crack tip. The Heaviside function (H) is used to model strong discontinuities such as cracks; in other words, it represents the displacement jump at the crack surface. The asymptotic crack tip functions ($F_{\alpha }$) represent the singularity near the crack tip while ${\underline{u}}_{\alpha }^{I}$ provides an additional degree of freedom to model and compute the crack propagation.

The partition of unity is an important characteristic of XFEM. As mentioned earlier, a damage model is required to model crack propagation using XFEM. In XFEM, the damage is modelled using either a cohesive law or fracture mechanics approach; both approaches are discussed below.

#### 4.1.1. XFEM Using Fracture Mechanics

Wang et al. [115] have developed a model for tear propagation in two-dimensional arteries using the energy-based approach, in which the linear elasticity based Griffith energy balance principle is extended to fibre-reinforced materials. The crack propagation criteria are based on the energy release rate G (ERR). It is defined as the variation in the total potential energy per unit propagation of the tear. Wang et al. [115] have numerically calculated ERR based on the variation in the global energy, where the numerical approximation of G is defined using the potential energy Π and change in the crack length δa as:

(83) $$G=-\frac{\delta \Pi }{\delta a}=-\frac{\Pi_{a+\delta a}-\Pi_{a}}{\delta a}$$

Here, potential energy $\Pi \;\left(a\right)=U_{e}-W$, $U_{e}$ is the equilibrium strain energy of the tissue, and W represents the work done due to load. The anisotropic hyperelastic tissue response is incorporated using strain energy density defined by the HGO model [34]. To obtain $\Pi \left(a\right)$, a boundary value problem is solved numerically for increasing crack lengths at intermittent points and energy values so obtained are interpolated with a cubic spline polynomial. This interpolation smoothly approximates $\Pi \left(a\right)$, which can be used to estimate ERR. The obtained values of ERR are used in simulating the two-dimensional crack propagation in arteries. This model ignores the plastic effects of tear propagation.

Karimi et al. [116] have modelled the initiation and propagation of a crack in the coronary artery to study the relation between rupture in the coronary artery and atherosclerosis. To achieve it, crack initiation and propagation in the healthy and atherosclerotic human coronary arterial walls were simulated with cracks placed circumferentially along the luminal and in the radial direction. Their model makes use of the virtual crack extension method (VCE) of XFEM and elastoplastic fracture mechanics criteria for crack initiation and propagation. In particular, they used an energy release rate criteria (J-integral) using nonlinear fracture mechanics of LS-DYNA [117]. The J-integral using the VCE is defined using a continuous weighting function q. It is defined on the surface of the body as zero and unity on the crack front nodes and interior of the body surface. In VCE, J-integral is given as:

(84) $$J=-\frac{1}{\Delta A_{c}}\int_{A}^{\;}\left[\left(W\delta_{1i}-\sigma_{ij}u_{j,1}\right)q_{,i}+\sigma_{ij}\varepsilon_{ij,1}^{0}q\right]dA$$

where $\Delta A_{c}$ is the virtual increase in crack area, W is the strain energy density, $\delta_{1i}$ is Kronecker delta, $\sigma_{ij}$ is stress in the defined area, $\varepsilon_{ij,1}^{0}$ is the initial strain, and $u_{i}$ is the displacements in the area of interest. The yield parameters for rupture simulation were drawn from the uniaxial tension experiments. Their study made use of the standard library of LS-DYNA, which was originally developed by Lindström et al. [117]. The material constitutive response for the healthy and atherosclerotic coronary arteries is assumed to be linear elastic.

#### 4.1.2. XFEM Using Cohesive Law

In XFEM, a traction law defines the criterion for crack initiation and propagation using cohesive law. The studies of Jayendran and Ruimi [118] and Wang et al. [119] used the linear traction separation law developed by Ferrara and Pandolfi [105]. Both studies simulate crack propagation in arteries, whereas the Wang et al. [119] study dealt with crack propagation in the residually stressed artery. The crack propagation is governed using linear traction separation law given as:

(85) $$G_{c}=\frac{1}{2}T_{c}\Delta u_{c}$$

where $G_{c}$ is the separation energy, $T_{c}$ is maximum traction before the damage and $\Delta u_{c}$ is the maximum displacement jump. $G_{c}$ and $\Delta u_{c}$ are the material parameters. When the maximum principal stresses ($\sigma_{p}$) reaches $T_{c}$, the displacement jump (Δu) is evaluated, and the crack propagates when $\Delta u>\Delta u_{c}$.

The study of Jayendran and Ruimi [118] aims to investigate the state of stresses in the artery during crack propagation, which would help study the mechanics of aortic dissections. In their study, a three-layer artery model simulated a radial tear in the intima layer and a circumferential tear in the media layer. Wang et al. [119] have used a two-layer arterial model with residual stresses to study their effect on propagating arterial dissections. The initial tear was placed circumferentially in the middle of the media loaded with internal pressure. Both the studies have used the anisotropic hyperelastic model of HGO [34] for the arteries. Additionally, both the studies authors have defined crucial parameters for CZM from uniaxial tension results by Holzapfel [75].

### 4.2. Cohesive Zone Modelling

In XFEM with cohesive law, a traction separation law is defined along the surface where crack propagates, which is not known as apriori. While in CZM, the traction separation defines the onset of crack and its propagation along the predefined crack propagation path. In CZM, a layer of the surface is placed between two bulk materials where the crack propagates. This layer of the surface is modelled with special elements that vanish when the crack propagates based on the traction separation and evolution. In this section, CZMs of the soft tissue is discussed.

Badel et al. [120] applied CZM to an atherosclerotic coronary artery to study dissection mechanisms triggered due to angioplasty. The simulation uses a two-dimensional model of the artery, i.e., partially embedded in the myocardium and epicardium. And the artery is modelled with two layers of medial with a plaque incorporated. Medial layer material response is modelled with a Neo-Hookean model, and epicardium and myocardium are modelled as a linear elastic material. The damage initiation criteria (DIC) for the onset of the material degradation in the interface is defined as:

(86) $$DIC=\max\left[\frac{\delta_{n}}{\delta_{n}^{0}},\frac{\delta_{t}}{\delta_{t}^{0}}\right]=1$$

where $\delta_{n}^{0}$ and $\delta_{t}^{0}$ are the maximum separation limits that define the damage initiation in normal and tangential directions, respectively.

The overall damage is characterized by D, a scalar damage variable that is specified on the onset of damage in the interface. D is a monotonically increasing variable from zero for without damage to one where the crack propagates. The effect of D on the contact stress components is given as:

(87) $$\sigma_{n}=\left\{\begin{array}{c} \left(1-D\right)\;\bar{\sigma_{n}},\;if\;\bar{\sigma_{n}}>0\\ \;\bar{\sigma_{n}},\;\;\;\;\;\;\;\;\;\;\;\;if\;\bar{\sigma_{n}}<0 \end{array}\right.$$

(88) $$\sigma_{t}=\left(1-D\right)\;\bar{\sigma_{t}}$$

where $\bar{\sigma_{n}}$ is normal and $\bar{\sigma_{t}}$ are tangential components of contact stress evaluated using elastic traction separation response without damage given as $\bar{\sigma_{i}}=Q_{i}\delta_{i}$. Here $\delta_{i}$ is the separation in the i direction and $Q_{i}$ is stiffness parameter (units is MPa/mm) that specifies the separation and interfacial stress. The damage variable D is defined as:

(89) $$D=\int_{\delta_{m}^{0}}^{\delta_{m}^{f}}\frac{\sigma_{n}d\delta_{n}+\sigma_{t}d\delta_{t}}{G_{c}-G_{0}}$$

where $G_{c}$ and $G_{0}$ are the critical fracture energy and elastic energy at damage initiation, respectively. And $\delta_{m}^{0}$ & $\delta_{m}^{f}$ are effective separation at the initiation of damage and propagation of the crack. Wherein the effective separation defined as $\delta_{m}=\sqrt{\delta_{n}^{2}+\delta_{t}^{2}\;}$. The critical parameters required for the traction separation are defined from the literature.

Leng et al. [18,121] have used CZM in two different studies applied to the artery. In one study, the fibrous cap delamination process is simulated to investigate its underlying process resulting in the delamination. Furthermore, the other study uses CZM to model the delamination in medial layers of the artery to study the failure mode. In both these studies, HGO [34] model is used to model the anisotropic hyperelastic response. Additionally, in Leng et al. [121] study, viscoelasticity is also considered. Effective displacement jump, δ, and effective traction, t are used to define the CZM, and they are given as:

(90) $$\delta =\sqrt{\lambda^{2}\left(\delta_{s1}^{2}+\delta_{s2}^{2}\right)+\delta_{n}^{2}}$$

(91) $$t=\sqrt{\lambda^{-2}\left(t_{s1}^{2}+t_{s2}^{2}\right)+t_{n}^{2}}$$

while $\delta_{s1}$ & $\delta_{s2}$ represents the shearing and tearing displacements in tangential directions of the cohesive surface, $\delta_{n}$ is the opening displacement, and λ is a scalar parameter that assigns weights to the displacements. Similarly, $t_{n}$, $t_{s1}$, and $t_{s2}$ are the tractions in normal and two shear directions across the cohesive surface.

In loading conditions, by using exponential CZM [106], the effective traction is given by

(92) $$t=e\sigma_{c}\frac{\delta }{\delta_{c}}\exp\left(-\frac{\delta }{\delta_{c}}\right),\;if\;\delta \leq \delta_{max}\;or\;\dot{\delta }\geq 0$$

(93) $$t_{n}=K\delta_{n},\;if\;\delta_{n}<0$$

Additionally, the effective traction during the unloading condition is given by:

(94) $$t=\left(\frac{t_{max}}{\delta_{max}}\right)\delta ,\;if\;\delta <\delta_{max}\;or\;\dot{\delta }<0$$

where $e=\exp\left(1\right)\approx 2.71828$, $\sigma_{c}$ is cohesive strength of the material, the maximum effective displacement is defined as $\delta_{c}=\frac{G_{c}}{e\sigma_{c}}$, $G_{c}$ is critical energy release rate, which is a material constant, K is the penalty stiffness of the penetration resistance, $\delta_{max}$ is the maximum effective displacement during one delamination cycle, and $t_{max}$ is the corresponding effective traction.

A scalar damage parameter  d is defined on the onset of the damage, which monotonically increases from zero for without damage to one where the crack propagates. The damage parameter based on the displacement jump function is defined as:

(95) $$d=1-\left(1+\frac{\delta_{max}}{\delta_{c}}\right)\exp\left(-\frac{\delta_{max}}{\delta_{c}}\right)$$

The crack initiates when $\delta =\delta_{c}$ resulting in the loss of the effective load-carrying capability of the cohesive elements. When $\delta =\delta_{sep}$, the material does not carry any load, and the crack propagates with the element deletion. Critical parameters required for their CZM are derived from the delamination experimental results.

Noble et al. [122] used CZM to simulate catheter-induced dissections (CID) in the porcine aorta. The tissue’s constitutive response was modelled with Ogden hyperelastic model given by:

(96) $$\Psi =\overset{2}{\underset{p=1}{\sum }}\frac{\mu_{p}}{\alpha_{p}}\left({\bar{\lambda }}_{1}^{\alpha_{p}}+{\bar{\lambda }}_{2}^{\alpha_{p}}+{\bar{\lambda }}_{3}^{\alpha_{p}}-3\right)+\frac{9}{2}K{\left(J^{\frac{1}{3}}-1\right)}^{2}$$

where $\mu_{p}$ is shear modulus and $\alpha_{p}$ are the dimensionless constants such that $\mu =\frac{1}{2}\overset{2}{\underset{p=1}{\sum }}\mu_{p}\alpha_{p}$. ${\bar{\lambda }}_{1},\;{\bar{\lambda }}_{2},\;{\bar{\lambda }}_{3}$ represents the principal stretches.

The cohesive zone given by Bosch et al. [123] was employed to account for the large deformations. Since the opening and traction vectors are evaluated globally, no distinction was made between normal and tangential directions. The traction vector t=te and separation vector δ=δe are related by:

(97) $$t=\frac{G_{c}}{\delta_{c}}\;\left(\frac{\delta }{\delta_{c}}\right)\exp{\left[-\frac{\delta }{\delta_{c}}\right]}^{\;}$$

where $\delta_{c}$ represents critical opening displacement, $G_{c}$ represents the critical energy release rate, and the unit vector e is specified along the line bounded by all points opposing the interface.

Critical traction $t_{max}$ for the material at the critical opening point (i.e., $\delta =\delta_{c}$), $t_{max}$ is given by:

(98) $$t_{max}=\frac{G_{c}}{\delta \exp\left(1\right)}$$

where $G_{c}$ is obtained from the experimental force-displacement graphs of the tissue dissection tests, whereas $\delta_{c}$ or ($t_{max}$) is found through the initiation zone of the same experimental force-displacement graphs [49]. Noble et al. [122] have conducted wedge dissection experiments for defining rupture parameters for the CZM. In their study, the crack was propagated by element deletion once the critical traction condition was achieved.

The CZM developed by Maiti and Geubelle [124] was applied to different soft tissue for tear studies. Fortunato et al. [125] applied this model to simulate tear propagation in arterial tissue during uniaxial testing. Ferrer et al. [126] used the model of Maiti and Geubelle to simulate tear propagation in tendons to study the effect of localized tendon remodelling. The traction separation law is normal ($t_{n}$) and tangential ($t_{t}$) direction across the cohesive surface is defined as:

(99) $$t_{n}=\frac{d}{1-d}\frac{\sigma_{max}}{d_{ini}}\;\left(\frac{\delta_{n}}{\delta_{nc}}\right)$$

(100) $$t_{t}=\frac{d}{1-d}\frac{\tau_{max}}{d_{ini}}\;\left(\frac{\delta_{t}}{\delta_{tc}}\right)$$

where $\sigma_{max}$ & $\tau_{max}$ are the maximum normal and shear stress of the cohesive zone, respectively, $\delta_{nc}$ & $\delta_{tc}$ are the critical displacement jumps corresponding to normal and tangential direction, $\delta_{n}$ & $\delta_{t}$ are the displacement jumps corresponding to the normal and tangential direction and d is the monotonically decreasing damage variable which is scalar and is defined as:

(101) $$d=\min\left[d_{ini},\;1-\delta \right]$$

here $d_{ini}$ is initially defined as damage parameter, Macauley bracket is defined as $1-\delta =\max\left(0,\;1-\delta \right)$ and the magnitude of displacement jump is defined as $\delta :=\sqrt{\delta_{n}^{2}+\delta_{t}^{2}}$. Both these studies used the HGO model to define an anisotropic hyperelastic material response and CZM parameters from the literature. The study of Ferrer et al. [126] has used only traction in the normal direction since the tissue is under uniaxial tension.

### 4.3. Crack Phase-Field Modelling

**Figure 8:**
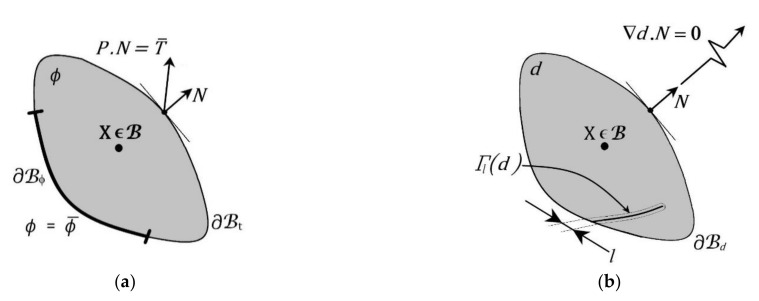
Multi-field problem: (**a**) Deformation with boundary conditions, (**b**) Crack phase-field with boundary conditions [Reprinted with permission from ref. [111], Copyright 2016 Elsevier].

The crack phase-field model (CPFM) defines the discontinuity with a special field equation along with the balance of linear momentum equation for the continuous field describing the elastic response of the material. A Ginzburg-Landau equation for defining the crack phase was introduced by Hakim and Karma [127]. Primarily two field variables are used in CPFM, i.e., the deformation map (φ) and crack phase-field (d), as shown in Figure 8a,b, respectively. The crack phase-field (d) is solved with the crack evolution equation (102), while the deformation field (φ) is solved with linear momentum balance (103).

The governing equations of CPFM problem to model fracture in anisotropic hyperelastic solid given as,

(102) $$J\;div\;\left(J^{-1}\tau \right)+\rho_{0}\bar{\gamma }=0$$

(103) $$\left(d-l^{2}\Delta d-\left(1-d\right)\bar{H}\right)\dot{d}=0$$

where the Jacobian is defined with deformation gradient F as J:=detF, $\rho_{0}$ is the density, $\bar{\gamma }$ is the prescribed body forces, l is the length scale parameter, and Kirchhoff stress tensor for the multi-field problem is defined as $\tau :=g\left(d\right)\tau_{0}$. Where $\tau_{0}$ is the stress tensor for the rupture free material and $g\left(d\right)$ is the monotonically diminishing quadratic function given by:

(104) $$g\left(d\right)={\left(1-d\right)}^{2}$$

with the boundary conditions that describe the evolution of the phase-field results in the degradation of the tissue as defined by:

(105) $$g^{\prime }\left(d\right)\leq 0$$

The degradation is ensured by the above condition, with $g\left(0\right)=1,\;g\left(1\right)=0$ acts as the limits for the flawless and torn state of the material, $g^{\prime }\left(1\right)=0$ illustrates a saturation as d→1.

The dimensionless crack driving function is given as:

(106) $$\bar{H}=\frac{\Psi_{0}}{g_{c}/l}$$

Two distinct energy-based failure measures for ground matrix and fibres are considered. Accordingly, the isotropic and anisotropic strain energies of the dimensionless crack driving function is decomposed as:

(107) $$\bar{H}={\bar{H}}^{iso}+{\bar{H}}^{ani}$$

(108) $${\bar{H}}^{iso}=\frac{U_{0}+\Psi_{0}^{iso}}{g_{c}^{iso}/l},\;{\bar{H}}^{ani}=\frac{\Psi_{0}^{\mathrm{ani}}}{g_{c}^{ani}/l}$$

where $g_{c}^{iso}/l$ is critical fracture energy of the ground matrix per length scale, similarly $g_{c}^{ani}/l$ is for the fibres. They are defined from the experimental results of Sommers et al. [128]. For example, the anisotropic hyperelastic constitutive model for the intact artery is additively decomposed into the isotropic part with a neo-Hookean, and an exponential form considers the contribution of fibres for the anisotropic part [24].

(109) $$\Psi_{0}=U_{0}+\Psi_{dev}+\Psi_{af1}+\Psi_{af2}$$

where

(110) $$U_{0}=\kappa \left(J-\ln J-1\right)$$

The deformation and crack phase-field are decoupled to subproblems with one-pass operator-splitting to solve the multi-field problem. The non-convex multi-field problem is divided into two convex sub-problems that are numerically simple to simulate when compared to the monolithic scheme. Gultekin et al. [34] have extended the approach by considering the fibre distribution to incorporate the anisotropy in the crack phase-field. Further, this model was applied to the artery peel test with different stress-based and energy-based criteria, and they showed that energy-based criteria are well suited for soft biological tissues [112].

## 5. Discussion

CDM was used to study all the damage phenomena, namely the Mullins effect, hysteresis, and permanent set. The CDM approach has been widely used to model damage phenomena across various tissues: artery, rectus sheath, ligament, annulus fibrosis, blood vessels, and ascending thoracic aortic aneurysm. Depending upon the tissue HGO model was used to define the collagen fibre structure, and appropriate strain energy was used to define the matrix material. Damage was introduced in the deviatoric part of the strain energy density in the form of Kachanov [129] based internal variable. Then, the equivalent strain concept of undamaged material by Simo and Ju [54] is used to define damage initiation. Damage propagation is phenomenologically modelled based on the behaviour of the tissue under supraphysiological loading. Based on the modelling of the targeted phenomenon, each reviewed model follows a specific approach.

To capture tear phenomena in soft tissues, a combination of CDM and SPH was used by Rausch et al. [67], and CDM combined with XFEM was proposed by Fathi et al. [69]. The model proposed by Ghasemi et al. [35] also captured soft tissue rupture. In addition, to circumvent the mesh dependency in CDM, a local gradient enhancement [66,74] and nonlocal integrals were applied [70,76]. While most of the reviewed models used a stretch-based damage evolution, Mousavi et al. [71] have used a fracture energy-based damage evolution. CDM models were developed in conjunction with various constitutive models, namely neo-Hookean, Ogden, and HGO. Additionally, Gao et al. [69] have developed a multi-physics model that considers water content in the constitutive model. While the damage model of Blanco et al. was developed from the mesoscopic level to model softening phenomena.

In pseudo-elasticity, the study of Pierce et al. [84] on the diseased and healthy aortic aneurysms using damage experiments have found a good agreement of their damage model. Wherein it demonstrates the capability of the pseudo-elasticity approach in modelling the softening and permanent. Further, the study of Holzapfel and Ogden [86] extends the pseudo-elasticity approach to consider the microstructural effect of collagen cross-links. Mainly, the effect of cross-links density and their interactions are considered, which can be physically interpreted as a soft tissue property. In addition to the softening and permanent set, Holzapfel and Ogden model [86] was able to capture the stiffening effect of the fibrous tissues with an increase in the density of the cross-links, while hyperelastic softening is a recently developed approach, and its application is limited to skin and arteries. The study of Li and Luo [63] has adopted the skin model and defined the parameters for swine, human, rabbit, and bovine skins. In a recent study, Volokh [88] has extended the initial model for two families of fibres, which can be applied to various soft tissue.

All damage modelling approaches are summarized in Table 3 with their capabilities, application to tissues, and benefits. In general, a stretch based criteria defined by Simo [67] is used to define damage initiation and its evolution. Mesh dependency can be surpassed by using a local gradient enhancement or nonlocal integral in damage evolution. In addition, CDM can be used for multi-physics problems and microstructural based problems. However, the number of damage parameters and their evolution equations increases the computation. The hyperelastic constitutive response is inherent when the HGO model is used for soft tissues, and it further elevates by damage. The fundamental behaviour of the damage model in pseudo-elasticity makes it a good fit for continuous and discontinuous softening, and it is also extended for the permanent set. Even though the model is straightforward for implementing numerical simulations, its application is limited to arteries and brain tissues. Particularly, Holzapfel and Ogden [86] damage model phenomenologically captures the damage with an additional physical parameter, i.e., cross-links. Pseudo-elasticity damage in the material is controlled by the maximum strain attained, making it numerically simple. Lastly, in softening hyperelasticity, the damage is incorporated in the constitutive model and does not involve any damage variables and their evolution equations. This approach is applied to model the permanent set in the artery and skin. As the model is still evolving, its capabilities and limitations are not fully explored.

Table 4 summarises the reviewed damage models, mechanisms and their validation methods. The authors of the reviewed models have validated with basic mechanical tests, which may differ from the physiological condition. For instance, damage models for rectus sheath are validated with the uniaxial tests by Martins et al. [72]. However, the biaxial tension would better represent the physiological loading condition in the rectus sheath. The same was mentioned as a limitation of the study by Martins et al. [72].Conventional mechanical tests can give the tissue essential mechanical properties. However, they may not behave similarly for the intended application [130]. Damage models developed for arterial tissues can be validated with internal pressure tests by Perez et al. [131]. The results are applicable in the domains such as balloon angioplasty and aneurysms. A damage model validated with physiological loading conditions would enable clinically translatable simulations.

For rupture modelling, most of the studies focused on the arterial tissues, while few were focused on the tendon. In XFEM, except for the study of Karimi et al. [116], rupture is simulated using a two-dimensional model with plane strain approximation. This approximation would be computationally efficient but oversimplifies the problem. While in the study of Karimi et al. [116], the virtual crack method of XFEM is used to simulate crack propagation in arteries. The CZM has been extensively used to study the delamination in arterial tissues. A good experimental agreement was reported in the reviewed studies for arterial delamination. Particularly, the CZM by Maiti and Geubelle [124] was used to study arterial rupture under uniaxial tension by Fortunato et al. [125], and the same model was applied for studying rupture in tendons by Ferrer et al. [126]. While XFEM is computationally expensive, CZM requires the crack path apriori. CPFM overcomes both the limitations of XFEM and CZM by dealing with rupture as a multi-field problem. A comparison of the three rupture approaches was reported by Gultekin et al. [33]. In CPFM, various stress-based and energy-based criteria can be used for crack initiation and propagation. However, as CPFM was recently adopted for soft tissues, its application is limited to arteries. Since CPFM uses multiple families of fibres and is given the freedom of using various crack initiation and propagation criteria, its implementation can be extended to other soft fibrous tissues.

The modelling of the damage parameter considers representing the damage in an inactive tissue by neglecting all the biological aspects of tissue [6,32]. In the discussed damage models, damage initiation is considered based upon the loading condition, in which the damage is initiated after reaching a particular load or particular stretch in the tissue caused by the load. Such models work to simulate the soft tissues under supra-physiological loading, i.e., when the tissues undergo loading higher than the physiological limit. In general, supra-physiological loading occurs due to external loading, for instance, in anterior cruciate ligament tear, catheter induced dissections, balloon angioplasty, etc. The damage model for diseased tissue requires knowledge of supra-physiological loads as well as pathological effects. However, these effects are not considered while modelling damage in diseased tissue.

Therefore, numerical simulation of the damage due to pathological conditions demands a model where its parameters represent the mechanical and physiological changes due to disease [6,139]. Such a model would be able to describe both the anatomical and physiological changes. Developing the aforementioned damage model requires constitutive model parameters with physical interpretation [32] and robust experiments in conjunction with tissue engineering to study the effect of disease on the tissue [139]. However, the active response of the soft tissues in damage and rupture models can be developed by introducing the growth and remodelling [140,141,142]. The damage models by Ghasemi et al. [36] considers damage mechanisms at mesoscopic scales, and Holzapfel and Ogden [86] consider the cross-links between the collagen fibres, where both the models aim towards defining the constitutive parameters with physical interpretation. So far, the studies reported are based on the experiments of healthy tissue and diseased tissue, which gives the constitutive response of the tissue in different conditions. However, the effect of the disease progression on the constitutive response is still at large. The advancements in tissue engineering and evolving in-vitro disease modelling [143] can enable the experiments to study the effect of disease progression [144] in the tissues.

## 6. Conclusions

A series of state-of-the-art damage and rupture models for modelling soft tissue failure were reviewed. The damage models were classified based on the approach of employing the damage in the soft tissues. Similarly, rupture models are grouped based on the method to deal with discontinuity during the rupture process. However, the present study has two limitations; firstly, it does not cover the modelling aspects of plastic phenomena (residual strains) and the capabilities of the discussed models to capture these phenomena. Secondly, the nanomechanics of the soft tissues to model damage and rupture were not reviewed.

In damage modelling, CDM and pseudoelasticity are widely applied to various tissues to model the damage under mechanical loading. Even though few microstructure-based damage models have been developed, there is a definite need for models that can consider the physiological effects of disease progression simulations. CDM based modelling uses reduction factor based on Kachanov [129] and equivalent strain concept of Simo and Ju [54], where it requires an initiation condition, evolution function for damage initiation and progression, respectively. However, in the pseudoelasticity, the damage variables are defined based on the maximum strain that occurred in the loading along with some damage control parameters. Lastly, the softening hyperelasticity approach uses an energy limiter incorporated in the strain energy density to model the softening effect. Further, it neither needs internal variables nor threshold conditions.

In rupture modelling, along with classical XFEM and CZM, recently developed CPFM is reviewed. CPFM overcomes complications of computations associated with XFEM and path dependency associated with CZM. However, the application of CPFM is limited to the artery, and its application to other tissues needs to be explored. The damage and rupture models reviewed here do not consider viscoelasticity, fibre recruitment, etc. These are considered as material effects and physiological effects. However, the reviewed damage models demonstrated their capability to capture different damage phenomena, and they were applied to various tissues in both humans and animals under supra-physiological loading. These damage and rupture models need to be extended to other tissues that can find biomedical and clinical research applications. For instance, simulation of the sutured condition of the vascular or skin grafts to evaluate the near in-vivo fracture toughness would help design artificial grafts. Further, the damage model of Gao et al. [70] can be extended to simulate ocular infections as this model captures the softening and tissue hydration. Moreover, corneal ectasia, where localised progressive softening occurs, can be studied by extending the model of Volokh to the cornea [88].

## Figures and Tables

**Table 1:** Summary of damage modelling techniques.

Modelling Considerations	Continuum Damage Mechanics	Pseudo-Elasticity	Softening Hyperelasticity
Strain energy density	$\Psi =\Psi_{vol}+\left(1-d\right)\left(\Psi_{dev}+\Psi_{ani}\right)$	$\Psi =\Psi_{vol}+\Psi_{dev}+\eta \Psi_{ani}+\phi \left(\eta \right)$	$\Psi =\phi \left[1-\exp\left(-\frac{W}{\phi }\right)\right]$
Damage parameter	$\mathrm{Kachanov}\;\mathrm{reduction}\;\mathrm{factor}\;\left(1-d\right)$	η–damage variable ϕ–damage function	ϕ–energy limiter
Damage initiation	$\mathrm{Equivalent}\;\mathrm{strain}\;\mathrm{based}\;\mathrm{on}\;\mathrm{Simo}\;\;\sqrt{2\Psi_{\alpha }}-r_{\alpha }\leq 0$ $r_{\alpha }$ –damage threshold at the current time.	Based on the critical stretch in fibres, i.e., $I_{i}\geq I_{ic},\;i=4.6$	Strain softening incorporated using energy limiters.
Damage evolution	Based on the model. Discontinuous damage modelled with the maximum strain in the loading path. Continuous damage is strain-rate-dependent	$\eta =1-\frac{1}{r}\mathrm{erf}\left[\frac{\Psi_{ani}^{max}-\Psi_{ani}}{m}\right]$ $\phi \left(\eta \right)=m\eta \log\eta +\left(1-\eta \right)\left[m+\Psi_{ani}\left(I_{ic}\right)\right]$	
Thermodynamic consideration	$\mathrm{Clausius}\text{–}\mathrm{Duham}\;\mathrm{inequality}\;\mathrm{used}\;\mathrm{to}\;\mathrm{define}\;\mathrm{damage}\;\mathrm{threshold}\;(r_{\alpha }$), which is maximum strain energy without damage.	In the primary loading curve η=1$\;\mathrm{and}\;\phi \left(1\right)=0$ and for the subsequent unloading and reloading, η<1$\;\mathrm{and}\;\phi \left(\eta \right)$ evolves, which is consistent with Clausius–Duham inequality.	Energy limiters activate the irreversible damage and dissipation that ensure the thermodynamic stability of the model.

**Table 2:** Summary of various damage models based on continuum damage mechanics (CDM).

References	Tissue	Tissue Structure	Damage	Modelling Features
Balanco et al. [64]	Soft tissue with fibres	Isotropic matrix and collagen fibres	Matrix and fibres	(1) Anisotropic, incompressible, (2) HGO strain energy function, (3) three parameters to define the damage, (4) continuum damage based on Simo and Ju [55].
Comellas et al. [65]	Rectus sheath	Isotropic matrix	Matrix	(1) Isotropic, incompressible, (2) strain energy function: neo-Hookean and Ogden, (3) one parameter related to the softening effect.
Polindara et al. [66]	Blood vessel	Isotropic matrix and collagen fibres	Fibres	(1) Anisotropic, incompressible, (2) HGO strain energy function, (3) two parameters to define the damage, (4) continuum damage based on Simo and Ju [55].
Ferreira et al. [67]	Arteries	Isotropic matrix and collagen fibres	Matrix and fibres	(1) Anisotropic, incompressible, (2) HGO strain energy function, (3) seven parameters to define the damage, (4) continuum damage based on Simo [68].
Rausch et al. [68]	Soft tissue with fibres	Isotropic matrix and collagen fibres	Matrix and fibres	(1) Anisotropic, incompressible, (2) HGO strain energy function, (3) two parameters to define the damage, (4) continuum damage based on Simo [68].
Fathi et al. [69]	Soft tissue with fibres	Isotropic matrix and collagen fibres	Matrix and fibres	(1) Anisotropic, incompressible, (2) HGO strain energy function, (3) six parameters to define the damage, (4) continuum damage based on Simo and Ju [55].
Gao et al. [70]	Annulus fibrosus	Isotropic matrix and collagen fibres	Matrix and fibres	(1) Anisotropic, incompressible, (2) HGO strain energy function, (3) four parameters to define the damage, (4) damage model is based on [38].
Mousavi et al. [71]	Ascending thoracic aortic aneurysms	Smooth muscle cells and collagen fibres distributed in the elastin matrix	Elastin matrix and collagen fibres	(1) Anisotropic, incompressible, (2) HGO strain energy function for fibres, (3) three parameters to define the damage, (4) damage model is based on linear softening by [66]
Ghasemi et al. [35]	Arteries	Elastin and collagen fibres in an isotropic matrix	Elastin fibres and collagen fibres	(1) Anisotropic, incompressible, (2) HGO strain energy function, (3) four parameters to define the damage, (4) continuum damage based on [58].

**Table 3:** Summary of damage modelling applications.

Damage Approach	Capabilities	Tissues	Benefits
CDM	Mullins effect Hysteresis Permanent set	Artery, Rectus sheath, ligament, annulus fibrosus, ascending aortic aneurysm, thoracic aneurysm.	Rupture can be simulated. Applied to multiphysics models. Applied across different constitutive models. Mesh-size independent.
Pseudo-elasticity	Mullins effect Hysteresis Permanent set	Aortic aneurysms, brain tissue	Less number of material parameters. Parameters with a physical meaning.
Softening hyperelasticity	Permanent set	Skin, artery	Damage is incorporated in the constitutive model. A simple model does not involve any internal variables and damage evolution equations.

**Table 4:** Damage models applied to tissues and their validation method.

Reference	Tissue	Mechanism	Validation
Blanco et al. [64]	Soft tissue	Mullins effect	Numerical simulation of tropocollagen failure by Buehler et al. [132]
Comellas et al. [65]	Rectus sheath	Mullins effect	Based on the uniaxial tension experiments of Martins et al. [72]
Polindara et al. [66]	Blood Vessel	Permanent set	Wedge geometry simulation for balloon angioplasty was validated with analytical of neo-Hookean tube tests [75].
Ferreira et al. [67]	Arteries	Mullins effect	The damage model is not validated.
Rausch et al. [68]	Arteries	Permanent set Rupture	Damage model with results of Stepmer et al. [133] and tear simulations with Tong et al. [134] and Sommer et al. [128]
Fathi et al. [69]	Rectus sheath Ligament	Mullins effect Rupture	Uniaxial tension experiments of Martins et al., [72] and numerical results of Waffenschmidt et al. [73] for rectus sheath. For ligament, the model is validated with experimental results of Weiss [77] and numerical results of Calvo et al. [135]
Gao et al. [72]	Annulus fibrosus	Permanent set	Simulation results validated with the experimental results of Ebara et al. [136] and Skaggs et al. [137]
Mousavi et al. [71]	Ascending thoracic aortic aneurysms	Permanent set	Buldge inflation test with graft size of 45 × 45 mm^2^ and inflation of circular area of diameter 30 mm.
Ghasemi et al. [35]	Arteries	Mullins effect Hysteresis Permanent set	Experiments of uniaxial tension tests and cyclic loading in uniaxial tension.
Pierce et al. [84]	Thoracic aortic tissues Abdominal aortic tissues	Permanent set Mullins effect	Experimental results from tissues under uniaxial tension and cyclic loading.
Holzapfel and Ogden [86]	Soft tissue	Mullins effect	A reduced model with uniaxial fibres is validated with the rat tail tendon experiment results of Pins and Silver.
Li and Luo [63]	Skin	Permanent set	Experimental results of Annaidh et al. [138] for human skin under uniaxial tension. Additionally, validate with porcine skin.
Volokh [88]	Artery adventitia	Permanent set	Uniaxial tension tests of artery adventitia in longitudinal and circumferential directions.

## Data Availability

Not applicable.
